# Epistatic effect of TLR3 and cGAS‐STING‐IKKε‐TBK1‐IFN signaling variants on colorectal cancer risk

**DOI:** 10.1002/cam4.2804

**Published:** 2019-12-23

**Authors:** Calogerina Catalano, Miguel Inacio da Silva Filho, Christoph Frank, Shun Lu, Katerina Jiraskova, Veronika Vymetalkova, Miroslav Levy, Vaclav Liska, Ondrej Vycital, Alessio Naccarati, Ludmila Vodickova, Kari Hemminki, Pavel Vodicka, Alexander N. R. Weber, Asta Försti

**Affiliations:** ^1^ Division of Molecular Genetic Epidemiology German Cancer Research Center (DKFZ) Heidelberg Germany; ^2^ Department of Internal Medicine V University of Heidelberg Heidelberg Germany; ^3^ Sichuan Cancer Center School of Medicine Sichuan Cancer Hospital & Institute University of Electronic Science and Technology of China Chengdu China; ^4^ Department of Molecular Biology of Cancer Institute of Experimental Medicine the Czech Academy of Sciences Prague Czech Republic; ^5^ 1st Medical Faculty Institute of Biology and Medical Genetics Charles University Prague Czech Republic; ^6^ Faculty of Medicine in Pilsen Biomedical Center Charles University Prague Pilsen Czech Republic; ^7^ First Medical Faculty Department of Surgery Charles University and Thomayer Hospital Prague Czech Republic; ^8^ Department of Surgery Teaching Hospital and Medical School of Charles University Pilsen Czech Republic; ^9^ Molecular and Genetic Epidemiology Italian Institute for Genomic Medicine (IIGM) Turin Italy; ^10^ Center for Primary Health Care Research Clinical Research Center Lund University Malmö Sweden; ^11^ Department of Immunology Interfaculty Institute for Cell Biology University of Tübingen Baden‐Württemberg Tübingen Germany; ^12^ Hopp Children's Cancer Center (KiTZ) Heidelberg Germany; ^13^ Division of Pediatric Neurooncology German Cancer Research Center (DKFZ) German Cancer Consortium (DKTK) Heidelberg Germany

**Keywords:** CRC, interaction, polygenic‐risk‐score, risk

## Abstract

**Objective:**

The TLR3/cGAS‐STING‐IFN signaling has recently been reported to be disturbed in colorectal cancer due to deregulated expression of the genes involved. Our study aimed to investigate the influence of potential regulatory variants in these genes on the risk of sporadic colorectal cancer (CRC) in a Czech cohort of 1424 CRC patients and 1114 healthy controls.

**Methods:**

The variants in the *TLR3, CGAS, TMEM173, IKBKE, and TBK1* genes were selected using various online bioinformatic tools, such as UCSC browser, HaploReg, Regulome DB, Gtex Portal, SIFT, PolyPhen2, and miRNA prediction tools.

**Results:**

Logistic regression analysis adjusted for age and sex detected a nominal association between CRC risk and three variants, *CGAS* rs72960018 (OR: 1.68, 95% CI: 1.11‐2.53, *P*‐value = .01), *CGAS* rs9352000 (OR: 2.02, 95% CI: 1.07‐3.84, *P*‐value = .03) and *TMEM173* rs13153461 (OR: 1.53, 95% CI: 1.03‐2.27, *P*‐value = .03). Their cumulative effect revealed a threefold increased CRC risk in carriers of 5‐6 risk alleles compared to those with 0‐2 risk alleles. Epistatic interactions between these genes and the previously genotyped *IFNAR1, IFNAR2, IFNA, IFNB, IFNK, IFNW, IRF3, and IRF7* genes, were computed to test their effect on CRC risk. Overall, we obtained nine pair‐wise interactions within and between the *CGAS, TMEM173, IKBKE*, and *TBK1* genes. Two of them remained statistically significant after Bonferroni correction. Additional 52 interactions were observed when IFN variants were added to the analysis.

**Conclusions:**

Our data suggest that epistatic interactions and a high number of risk alleles may play an important role in CRC carcinogenesis, offering novel biological understanding for the CRC management.

## Graphical abstract

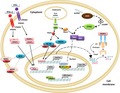

## 1 INTRODUCTION

Colorectal cancer (CRC) is the third most common cancer and the fourth leading cause of cancer mortality worldwide, with an estimate of 1.8 million new cases and close to 1 million deaths in 2018.^1^ It originates from the epithelial cells lining the colorectal tract, as a consequence of the gradual accumulation of epigenetic and genetic alterations that lead to the transformation of physiological colonic mucosa to adenocarcinoma.^2^ About 85% of CRCs are sporadic and occur in people that have no family history of CRC.^3^

So far, genome‐wide association studies have reported ~100 risk loci for CRC highlighting new genes and pathways contributing to CRC susceptibility and suggesting roles for Hedgehog signaling, Krüppel‐like factors, Hippo‐YAP signaling, and immune function.^4^, ^5^ Hua et al have also suggested that polymorphisms within xeroderma pigmentosum group C (XPC) and G (XPG) genes may affect CRC susceptibility through impairment of the nucleotide excision repair (NER) pathway.^6^, ^7^

Moreover, chronic intestinal inflammation has long been recognized as a prominent CRC driver.^8^ Patients affected by inflammatory bowel diseases (IBD), such as Crohn's disease or ulcerative colitis, have been reported to have an increased risk of CRC development.^9^ Another factor modulating CRC risk appears to be the intestinal microbiota, the plethora of microorganisms populating the human intestine.^10^ The immune system plays an important role in keeping the balance between commensalism, harmful pathogen elimination and self‐tolerant maintenance; a disruption of this balance greatly contributes to chronic inflammation.^11^, ^12^ In this scenario, a pivotal role is played by the pattern recognition receptors (PRRs), among which Toll‐like receptors (TLRs) are able to recognize different microbe‐associated molecular patterns (MAMPs) and/or damage‐associated molecular patterns (DAMPs), induce expression of several cytokines, and stimulate activation and differentiation of dendritic cells (DCs). Especially, TLR3, TLR7, and TLR9 are able to stimulate both interferon α (IFNα) and IFNβ.^13^ Focusing on TLR3, it is able to recognize viral dsRNA and to activate mitogen‐activated protein kinase 1 (MAPK), nuclear factor‐κB (NF‐kB) and type I IFN signaling pathways through TIR domain‐containing adaptor‐inducing interferon‐β (TRIF), leading to the production of chemokines and cytokines, such as IL‐1β, IL‐6, and TNFα. Particularly, TLR3 uses the TRIF ‐ TNF Receptor Associated Factor 3 (TRAF3) ‐TANK‐binding kinase 1 (TBK1) + Inhibitor of nuclear factor kappa‐B kinase subunit epsilon (IKKε)—Interferon regulatory factor 3 (IRF3) axis to trigger IFNβ and antiviral responses.^14^, ^15^ Moreover, many studies have reported that TLR3 signaling is not only able to induce type I IFN pathways, but indirectly also a strong CD8^+^ T cell response. Indeed, TLR3 induces a cross‐presentation of cell‐associated antigens, pivotal for cytotoxic T lymphocyte induction, implying an important role in starting adaptive immune responses.^16^, ^17^ The same IKKε‐TBK1‐IRF3 axis is used by cyclic‐GMP‐AMP synthase (cGAS), which can be activated by the recognition of cytosolic DNA, derived either from pathogens or self‐DNA.^18^ Once activated, cGAS activates stimulator of interferon genes protein (STING) (encoded by (transmembrane protein 173 (*TMEM173*)) via the cyclic‐adenosine‐guanosine‐monophosphate (cGAMP) second messenger to activate the TBK1‐IRF3‐dependent signaling. IRF3 phosphorylation and nuclear translocation then triggers the type I IFN response^18^ (Figure S1). Recently, a deregulation of these pathways in CRC has been reported, mainly caused by an imbalanced expression of the coding genes.^19^ Impaired expression of STING has been revealed to favor persistent inflammation and allow the tumor to evade immunosurveillance, thus laying the foundation for tumor initiation and progression.^20^ *TLR3* expression in CRC is quite controversial; indeed, while Nojiri et al reported a similar expression pattern between non‐malignant epithelial and colon carcinoma cells,^21^ Niedzielska et al reported an inversely proportional relation between *TLR3* expression level and malignancy stage.^22^, ^23^ On the other hand, germline variation on *TLR3* has been associated with poor prognosis.^24^

To shed light on the potential role of the single nucleotide polymorphisms (SNPs) within the *TLR3, CGAS, TMEM173*, *TBK1,* and *IKBKE* genes, we genotyped a set of 11 potential regulatory SNPs in a case‐control study of 1424 CRC patients and 1114 healthy controls from the Czech Republic and evaluated their association with CRC risk. Moreover, we investigated whether their combined effect and/or pair‐wise interactions between all the evaluated SNPs and the previously genotyped SNPs in the *IFNA, IFNB, IFNK, IFNW1, IRF3, IRF7, and IFNAR1/2* genes may influence CRC risk.^25^

## 2 MATERIALS AND METHODS

### 2.1 Ethics statement

The ethical approval for this study design was obtained from the Institute of Experimental Medicine Academy of Sciences of the Czech Republic (Prague, Czech Republic) and the Institute for Clinical and Experimental Medicine and Faculty Thomayer Hospital (Prague, Czech Republic). Written informed consent was signed by each participant in accordance with the Helsinki declaration.

### 2.2 Study population

Details of the studied populations are described elsewhere.^26^ Briefly, the case group contained 1424 CRC patients recruited between the years 2004 and 2013 by several oncological departments in the Czech Republic (Table 1). Their mean age was 62.7 years, and 61.8% of them were men. The patients showed positive colonoscopic results for malignancy, histologically confirmed as colon or rectal carcinomas. Patients with any previous history of cancer or those who met the Amsterdam criteria I or II for hereditary nonpolyposis colorectal cancer were not included in the study. The control group contained 1114 healthy individuals recruited by the blood‐donor centers in Kralovske Vinohrady Hospital and Vojkov hospital in Prague.^27^ Their mean age was 47.1 years, and 53.4% of them were men. Other characteristics, such as smoking, drinking status and body mass index were not available for the vast majority of the individuals, therefore none of them was taken into consideration in the analysis.

**Table 1:** Characteristics of the study population

CRC risk analysis	Cases	Controls	*P*‐value
All patients	1424	1114	
Age at diagnosis			
Mean (range)	62.7 (24‐90)	47.1 (18‐94)	**<.0001** a
Median	63	47	
Sex			
Male	880 (61.8%)	595 (53.4%)	**2.6e‐05** b
Female	544 (38.2%)	519 (46.6%)	
Tumor location			
Colon	889 (62.4%)		
Rectum	398 (27.9%)		
Missing information	137 (9.6%)		

Note

Significant results are in bold.

a Z statistics: Wilcoxon Rank‐Summ‐Test

b Chi‐square.

### 2.3 SNP selection

A total of 11 common SNPs (minor allele frequency, MAF ≥ 0.10 in the CEU population), with a pairwise linkage disequilibrium (LD) *r* ^2^ ≤ .80, were selected for genotyping within five genes, namely *TLR3*, *CGAS*, *TMEM173*, *IKBKE,* and *TBK1*. Candidate SNPs were non‐coding SNPs located within the 5ʹ flanking region, 5′ and/or 3′ untranslated regions (UTRs) or they were expression quantitative trait loci (eQTL) SNPs for the selected genes or non‐synonymous SNPs, validated by 1000 Genomes in the CEU population (Table S1).

Additionally, a total of 24 potentially functional SNPs within promoter, or 5ʹUTR or 3ʹUTR of the genes involved in the IFN signaling pathway, including *IFNA (1, 2, 4, 5, 7, 8, 13, 16, 17, and 21), IFNB1, IFNK, IFNW1, IRF3, IRF7, and IFNAR1/2,* were selected from our previous study^25^ (Table S2). Notably, *IFNAs, IFNB1, IFNK, IFNW1* genes are all located at the same chromosome location (9p21.3), and capture many other SNPs in linkage, supplying further information on other genes at the given locus.

### 2.4 In‐silico analysis

Online bioinformatic tools were used to explore and select the SNPs of interest, including UCSC browser (https://genome-euro.ucsc.edu/), HaploReg http://www.broadinstitute.org/mammals/haploreg/haploreg.php)*,* Regulome DB (http://www.regulomedb.org/), Gtex Portal (https://gtexportal.org/home/), MicroSNiPer (http://epicenter.iefreiburg.mpg.de/services/microsniper/) SIFT (Sorting Intolerant from Tolerant) (http://sift.jcvi.org/) and PolyPhen2 (Polymorphism Phenotyping v2) (http://genetics.bwh.harvard.edu/pph2/). LD and haplotype blocks within the genes were examined based on pairwise LD *r* ^2^ (Table S1).

### 2.5 Genotyping

SNP genotyping was performed on genomic DNA from peripheral blood leukocytes using KASP (LGC genomics, Hoddesdon, Hertfordshire, UK) and TaqMan (Thermo Fisher Scientific) as allelic discrimination methods. DNA amplification was carried out in accordance with the LGC genomics’ and TaqMan's PCR cycling conditions. Genotype detection was performed using the ViiA 7 Real‐Time PCR System (Thermo Fisher Scientific), and setting the range 94.0%‐100% as a threshold for the genotype call rate. The genotype correlation between the 142 duplicated samples, used as quality controls, was higher than 95%. Samples with <50% call rate over all assays were excluded from the study, leaving 1396 cases and 1111 controls for the association analysis.

### 2.6 Statistical analysis

The chi‐square test was performed to test the deviation of genotype frequencies in the controls from Hardy‐Weinberg equilibrium (HWE). Logistic regression analysis adjusted for age and sex was used to calculate odds ratios (ORs) and 95% confidence intervals (CIs) for associations between genotypes and CRC risk (SAS Version 9.3; SAS Institute).

In the combined analysis of the three SNPs that showed a nominal association with CRC, the allelic model was calculated for each SNP whereby the genotypes were converted into 0, 1, and 2 risk alleles. On the basis of the number of risk alleles, a genotype score ranging from 0 to 6 was constructed. Samples with one or more missing genotypes were not included.

To evaluate the significant findings, the false‐positive report probability (FPRP) was calculated.^28^ A prior probability of 0.1 and an FPRP threshold of 0.2 were assigned to detect an OR of 0.67/1.50 (protective/risk effects) for the association with genotypes and alleles numbers under investigation. Only the associations with an FPRP value less than 0.2 were considered noteworthy findings.

Binary interactions for all different SNP combinations were evaluated to investigate whether the non‐additive effect can improve the prediction of the disease risk. The newly genotyped SNPs were complemented and analyzed with the SNPs in the *IFNA, IFNB, IFNK*, *IFNW1, IRF3, IRF7,* and *IFNAR1/2* genes previously genotyped in a subset of 1327 CRC patients and 758 controls from the same Czech cohort.^25^ Details of the pair‐wise interaction analyses are described elsewhere.^26^ Briefly, four different modes of inheritance were calculated and tested for each pair: the three genotypes model, the log‐additive model, the dominant model, and the recessive model. To assess whether the SNP‐SNP interaction term led to a considerably better fit of the data, likelihood ratio tests were performed. The best model for SNPs that showed significant interactions with each other by more than one model was selected on the basis of their Akaike information criterion (AIC). The smaller the value of AIC, the better the model data fit. To evaluate the benefit of all genetic components (including SNPs and the interaction term) to the model, likelihood ratio test‐based P‐values were calculated. The corresponding ORs and the Wald estimate for their 95% CIs and P‐values were computed for the best model of each SNP pair. As the reference genotype combination we used the major allele genotype combination based on the best model of each interaction. Altogether, 55 (11 SNPs*(11‐1) /2) independent tests were performed between the *TLR3, CGAS, TMEM173, IKBKE, and TBK1* genes, giving a Bonferroni corrected p‐value of 0.05/55 = 0.0009. Inclusion of the IFN pathway genes to the study increased the number of independent tests to 275, giving a Bonferroni corrected *P*‐value of 0.05/275 = 0.0002.

## 3 RESULTS

### 3.1 Single SNP analysis

The minor allele frequencies of the genotyped SNPs were similar to the ones reported in the European population in the 1000 Genomes Project (http://www.internationalgenome.org/) and in the non‐Finnish European population in the Genome Aggregation Database (https://gnomad.broadinstitute.org/).

The genotype distribution of all SNPs was consistent with HWE in the control group (*P* > .05). Three SNPs, two located within ***CGAS***, rs72960018 (OR: 1.68, 95% CI: 1.11‐2.53, *P*‐value = .01, under dominant model) and rs9352000 (OR: 2.02, 95% CI: 1.07‐3.84, *P*‐value = .03, under recessive model), and one within ***TMEM173***, rs13153461 (OR: 1.53, 95% CI: 1.03‐2.27, *P*‐value = .03, under recessive model), exhibited moderate associations with CRC risk (Table 2). However, when considering an FPRP threshold of 0.2, none of them was considered a noteworthy finding (Table S3).

**Table 2:** Association of single SNPs with CRC risk

Gene ID	SNP ID	Genotype	Cases	Controls	OR	95% CI	*P*
*CGAS*	rs72960018	A/A	69	70	1.00		
		A/G	505	408	1.6	(1.04‐2.46)	**.03**
		G/G	789	614	1.73	(1.13‐2.63)	**.01**
		A/A	69	70	1.00		
		A/G + G/G	1294	1022	1.68	(1.11‐2.53)	**.01**
	rs9352000	T/T	935	761	1.00		
		G/T	323	237	1.03	(0.82‐1.30)	.77
		G/G	44	19	2.04	(1.07‐3.88)	**.03**
		T/T + G/T	1258	998	1.00		
		G/G	44	19	2.02	(1.07‐3.84)	**.03**
	rs34413328	A/A	854	666	1.00		
		A/‐	452	377	0.97	(0.79‐1.19)	.75
		−/−	56	49	1.1	(0.68‐1.80)	.69
	rs610913	T/T	499	387	1.00		
		G/T	617	521	0.91	(0.73‐1.12)	.37
		G/G	228	171	1.13	(0.85‐1.51)	.41
*TMEM173*	rs7380272	C/C	1053	887	1.00		
		C/T	283	199	1.21	(0.94‐1.55)	.13
		T/T	28	15	1.57	(0.75‐3.26)	.23
	rs13153461	A/A	751	646	1.00		
					
		A/G	509	396	1.12	(0.91‐1.38)	.28
		G/G	106	60	1.6	(1.07‐2.39)	**.02**
		A/A + A/G	1260	1042	1.00		
		GG	106	60	1.53	(1.03‐2.27)	**.03**
*IKBKE*	rs2297549	T/T	816	663	1.00		
		C/T	481	387	0.97	(0.79‐1.19)	.75
		C/C	71	42	1.35	(0.84‐2.19)	.22
	rs2297548	T/T	890	752	1.00		
		C/T	419	303	1.2	(0.97‐1.49)	.10
		C/C	56	39	1.43	(0.86‐2.38)	.17
	rs15672	G/G	378	302	1.00		
		G/A	676	510	1.15	(0.91‐1.45)	.23
		A/A	276	265	0.87	(0.66‐1.15)	.33
*TBK1*	rs61933195	C/C	1007	819	1.00		
		A/C	319	262	0.9	(0.72‐1.13)	.37
		A/A	35	19	1.22	(0.61‐2.42)	.57
*TLR3*	rs3775291	C/C	663	512	1.00		
		C/T	573	479	0.92	(0.75‐1.13)	.42
		T/T	115	113	0.73	(0.52‐1.04)	.08

Note

Significant results are highlighted in bold.

Abbreviations: 95% CI: 95% Confidence Interval; OR, Odds Ratio; *P*, *P*‐value.

### 3.2 Combined analysis

Since *CGAS* and *TMEM173* encode proteins that are interacting with each other through a second messenger, cGAMP, we further estimated the cumulative effect of the three SNPs reporting a nominal association with CRC susceptibility. Patients in the highest risk score group (5‐6 risk alleles) had about threefold augmented risk of developing CRC compared to those in the lowest risk score group (0‐2 risk alleles) (adjusted OR = 2.98, 95%CI: 1.35‐6.56, *P* for trend: 6 × 10^−4^) (Table 3). An FPRP value less than 0.2 was observed for the score group containing individuals carrying 3‐4 risk alleles, but not for the highest risk score group, which showed also a low statistical power (Table S3). This suggests some possible bias in the findings due to reduced sample size, which need to be further validated in larger studies. Interestingly, no synergistic interaction was observed between these SNPs (data not shown).

**Table 3:** Combined risk analysis of *CGAS*, rs72960018 and rs9352000, and *TMEM173* rs13153461

Number of the risk alleles	Cases	Controls	OR (95% CI)	*P*
0‐2	705	624	1.00	
3‐4	512	362	1.31 (1.06‐ 1.62)	**.01**
5‐6	35	12	2.98 (1.35‐ 6.56)	**.007**
*P*‐trend = .0006				

Note

Significant results are highlighted in bold.

Abbreviations: 95% CI: 95% Confidence Interval; OR, Odds Ratio; *P*, *P*‐value.

### 3.3 SNP‐SNP interactions in CRC risk

We further evaluated whether a synergistic effect of the 11 SNPs within *TLR3, CGAS, TMEM173, TBK1,* and *IKBKE* genes may impact CRC risk. After setting our significance level of *P*‐value < .05, nine interactions, counting interactions between SNPs both within a gene and between the five genes, were observed (Table 4, Figure S2). Two out of nine interactions, ***IKBKE* rs2297549** ‐***TMEM173* rs13153461** and ***IKBKE* rs2297549 ‐ *TMEM173* rs7380272**, passed the Bonferroni correction (*P*‐value < .0009). The association with the risk of CRC was estimated for the best model of each SNP‐SNP interaction (Table S4).

**Table 4:** Pair‐wise interactions of *TLR3, CGAS, TMEM173, TBK1,* and *IKBKE* genotypes with cases and controls

Variable 1			Variable 2			Interaction			SNP total		
Gene	SNP	Mode of Inheritance	Gene	SNP	Mode of Inheritance	LRT Statistic	DG	*P* value	LRT Statistic	DG	*P *value
*IKBKE*	rs2297549	Recessive	*TMEM173*	rs7380272	Allele Number	13.13	1	.0003	18.44	3	.0004
*IKBKE*	rs2297549	Recessive	*TMEM173*	rs13153461	Recessive	11.69	1	.0006	16.90	3	.0007
*IKBKE*	rs15672	Allele Number	*TMEM173*	rs13153461	Allele Number	4.41	1	.036	8.87	3	.031
*IKBKE*	rs15672	Recessive	*TMEM173*	rs7380272	Dominant	4.48	1	.034	9.67	3	.022
*IKBKE*	rs2297549	Three Genotypes	*CGAS*	rs34413328	Dominant	12.08	2	.002	13.90	5	.016
*IKBKE*	rs2297548	Recessive	*TMEM173*	rs7380272	Dominant	7.14	1	.008	10.54	3	.015
*IKBKE*	rs2297549	Dominant	*CGAS*	rs72960018	Recessive	5.86	1	.016	10.01	3	.018
*TMEM173*	rs7380272	Allele Number	*CGAS*	rs9352000	Recessive	5.58	1	.018	13.34	3	.004
*TBK1*	rs61933195	Dominant	*CGAS*	rs9352000	Recessive	4.21	1	.04	11.87	3	.008

Abbreviations: DG, Degrees of Freedom; LRT, Likelihood Ratio Test.

The two ***IKBKE*** SNPs, **rs2297549** and **rs15672** (*r* ^2^ = .01), showed an interesting and complex interaction with the same two ***TMEM173*** SNPs, **rs13153461** and **rs7380272** (*r* ^2^ = .38). An increased CRC risk was observed particularly between ***IKBKE* rs2297549** and the two ***TMEM173*** SNPs when the minor allele homozygote genotypes of one gene interacted with the major allele containing genotypes of the other gene. On the other hand, an increased and decreased risk of CRC was observed when ***IKBKE* rs15672** (minor allele homozygote genotype) interacted with ***TMEM173* rs13153461** (minor allele homozygote genotype) and ***TMEM173* rs7380272** (major allele homozygote genotype)**,** respectively (Table S4).

### 3.4 SNP‐SNP interactions in CRC risk including the IFN variants

The two *TMEM173* SNPs were not only shown to be the main interaction partners within our candidate genes but also exhibited an interaction with many of the previously genotyped *IFN* variants (Table 5). Especially, ***TMEM173* rs13153461** showed four more interactions with ***IRF3* rs2304204**, ***IRF7* rs1061502**, ***IFNB1* rs1424855**, and ***IFNK* rs700782**, which are not in LD with each other. Compared to the reference genotype pair, an increased risk was observed for specific genotype pairs when ***TMEM173* rs13153461** interacted with the ***IRF3*, *IFNB1*,** and ***IFNK*** SNPs (Table S5). No significant ORs were detected for the ***IRF7*** interaction**.** On the other hand, ***TMEM173* rs7380272** showed interactions with another set of three *IFN* SNPs, ***IFNA7/14* rs6475526**, ***IFNA16*** **rs10964912,** and ***IFNA21* rs12376071**, which were in moderate LD with each other (*r* ^2^ = .40‐.50). A strong interaction was reported between ***TMEM173* rs7380272** (major homozygote genotype) and ***IFNA7/14* rs6475526** (minor allele genotypes). The other interactions were more complex and depended on the genotype combinations (Table S5, Figure S4).

**Table 5:** Pair‐wise interactions of genotypes *TLR3, CGAS, TMEM173, TBK1, IKBKE* and IFN genes genotypes with cases and controls

Variable 1			Variable 2				Interaction			SNP total		
Gene	SNP	Mode of Inheritance	Gene		SNP	Mode of Inheritance	LRT Statistic	DG	*P*‐value	LRT Statistic	DG	*P*‐value
*TMEM173*	rs13153461	Recessive	*IRF3*		rs2304204	Three Genotypes	8.62	2	.014	17.28	5	.004
*TMEM173*	rs13153461	Three Genotypes	*IRF7*		rs1061502	Dominant	7.08	2	.029	11.52	5	.042
*TMEM173*	rs13153461	Recessive	*IFNB1*		rs1424855	Three Genotypes	7.19	2	.028	11.97	5	.035
*TMEM173*	rs13153461	Recessive	*IFNK*		rs700782	Dominant	3.99	1	.046	12.58	3	.006
*IFNA7/IFN14*	rs6475526	Allele Number	*TMEM173*		rs7380272	Allele Number	4.13	1	.042	9.40	3	.025
*IFNA16*	rs10964912	Dominant	*TMEM173*		rs7380272	Recessive	5.46	1	.019	9.03	3	.029
*IFNA21*	rs12376071	Allele Number	*TMEM173*		rs7380272	Recessive	7.45	1	.006	9.65	3	.022
*IFNA4*	rs2383183	Dominant	*CGAS*		rs72960018	Allele Number	6.32	1	.012	11.48	3	.009
*IFNA4*	rs2383183	Dominant	*CGAS*		rs610913	Recessive	4.83	1	.028	8.93	3	.030
*IFNA13*	rs641734	Dominant	*CGAS*		rs72960018	Three Genotypes	7.40	2	.025	14.60	5	.012
*IFNA13*	rs641734	Dominant	*CGAS*		rs610913	Allele Number	3.90	1	.048	8.60	3	.035
*CGAS*	rs34413328	Dominant	*IFNK*		rs700782	Dominant	6.59	1	.010	10.32	3	.016
*CGAS*	rs610913	Allele Number	*IFNK*		rs700782	Dominant	4.92	1	.027	11.35	3	.010
*CGAS*	rs34413328	Allele Number	*IFNA7/IFN14*		rs6475526	Dominant	4.53	1	.033	9.54	3	.023
*CGAS*	rs610913	Allele Number	*IFNA7/IFN14*		rs6475526	Allele Number	4.04	1	.045	10.33	3	.016
*IFNA2*	rs10120977	Dominant	*CGAS*		rs9352000	Recessive	4.08	1	.043	10.36	3	.016
*IFNA2*	rs10120977	Dominant	*CGAS*		rs610913	Recessive	5.74	1	.017	8.93	3	.030
*IFNA16*	rs10964912	Dominant	*CGAS*		rs9352000	Recessive	6.23	1	.013	15.07	3	.002
*IFNA16*	rs10964912	Dominant	*CGAS*		rs610913	Recessive	4.34	1	.037	9.86	3	.020
*IFNAR2*	rs1131668	Dominant	*CGAS*		rs9352000	Recessive	5.92	1	.015	9.22	3	.027
*IFNAR2*	rs1131668	Allele Number	*CGAS*		rs610913	Recessive	6.24	1	.013	8.48	3	.037
*IFNK*	rs700782	Three Genotypes	*IKBKE*		rs2297548	Dominant	6.76	2	.034	12.52	5	.028
*IFNK*	rs700782	Three Genotypes	*IKBKE*		rs2297549	Dominant	6.33	2	.042	10.96	5	.05
*IFNAR1*	rs2834202	Dominant	*IKBKE*		rs2297548	Recessive	4.15	1	.042	13.11	3	.004
*IFNAR1*	rs2834202	Allele Number	*IKBKE*		rs2297549	Dominant	6.92	1	.009	8.85	3	.031
*IFNAR1*	rs2856968	Dominant	*IKBKE*		rs15672	Dominant	8.40	1	.004	14.72	3	.002
*IFNAR1*	rs2856968	Three Genotypes	*IKBKE*		rs2297549	Three Genotypes	14.25	4	.007	22.65	8	.004
*IFNA5*	rs12156640	Dominant	*TBK1*	rs61933195		Dominant	11.86	1	.0006	11.86	3	.008
*IRF3*	rs2304204	Dominant	*CGAS*	rs9352000		Recessive	6.22	1	.013	11.32	3	.010
*IFNAR2*	rs1131668	Allele Number	*TLR3*	rs3775291		Recessive	11.10	1	.0009	13.44	3	.004
*IFNAR1*	rs2257167	Dominant	*IKBKE*	rs15672		Allele Number	9.88	1	.002	11.84	3	.008
*CGAS*	rs72960018	Dominant	*IFNA17*	rs7873404		Three Genotypes	8.17	2	.017	13.54	5	.019
*IFNA21*	rs2939	Dominant	*CGAS*	rs610913		Allele Number	5.38	1	.020	9.49	3	.023
*IFNAR1*	rs2850015	Allele Number	*TLR3*	rs3775291		Recessive	5.31	1	.021	8.31	3	.040
*IFNB1*	rs1424855	Allele Number	*TBK1*	rs61933195		Dominant	8.87	1	.003	8.90	3	.031
*IFNA21*	rs2939	Dominant	*IKBKE*	rs2297548		Allele Number	5.03	1	.025	9.83	3	.020
*IFNAR1*	rs2856968	Allele Number	*CGAS*	rs72960018		Dominant	7.68	1	.006	16.93	3	.001
*IFNA7/IFN14*	rs6475526	Dominant	*TBK1*	rs61933195		Allele Number	4.84	1	.028	11.07	3	.011
*IFNAR1*	rs2834202	Three Genotypes	*CGAS*	rs610913		Recessive	10.31	2	.006	15.25	5	.009
*IFNA8*	rs10811536	Three Genotypes	*IKBKE*	rs2297549		Recessive	7.12	2	.028	12.15	5	.033
*IFNW1*	rs10757189	Dominant	*TBK1*	rs61933195		Dominant	7.55	1	.006	8.74	3	.033
*IFNA21*	rs2939	Dominant	*CGAS*	rs72960018		Dominant	7.54	1	.006	12.79	3	.005
*IFNAR1*	rs2856968	Recessive	*CGAS*	rs9352000		Recessive	4.44	1	.035	8.63	3	.035
*IFNAR1*	rs2856968	Three Genotypes	*CGAS*	rs610913		Recessive	10.12	2	.006	20.42	5	.001
*IFNA4*	rs2383183	Dominant	*IKBKE*	rs2297548		Allele Number	4.33	1	.037	8.04	3	.045
*IFNAR1*	rs2850015	Dominant	*CGAS*	rs72960018		Three Genotypes	6.57	2	.038	11.34	5	.045
*IRF7*	rs1061502	Recessive	*TLR3*	rs3775291		Three Genotypes	9.77	2	.008	13.95	5	.016
*IFNA21*	rs12376071	Dominant	*CGAS*	rs9352000		Recessive	7.03	1	.008	13.06	3	.005
*IFNW1*	rs10757189	Recessive	*CGAS*	rs9352000		Recessive	6.84	1	.009	11.69	3	.009
*IFNAR2*	rs1131668	Recessive	*IKBKE*	rs2297548		Recessive	6.78	1	.009	9.25	3	.026
*TLR3*	rs3775291	Dominant	*IFNA13*	rs641734		Dominant	3.90	1	.048	8.62	3	.035
*IFNAR1*	rs2257167	Allele Number	*CGAS*	rs72960018		Dominant	6.60	1	.010	11.75	3	.008

Abbreviations: DG, Degrees of Freedom; LRT, Likelihood Ratio Test.

The highest number of interactions was represented by the four ***CGAS*** SNPs, **rs72960018** (n = 8)**, rs9352000** (n = 9)**, rs34413328** (n = 3)**,** and **rs610913** n = 10) when analyzed in interplay with the previously genotyped *IFN* variants (Table 5, Figure S5). The unlinked SNPs, **rs72960018, rs9352000, rs34413328** (*r* ^2^ < .08) shared several interactions with **rs610913,** which was in a moderate LD with the other SNPs (*r* ^2^ = .20‐.38). Especially, we observed a decreased risk of CRC development when ***CGAS* rs610913** and **rs72960018** interacted with ***IFNA4* rs2383183** and ***IFNA13* rs641734** (*r* ^2^ = .43). Many genotype combinations of the ***CGAS*** SNPs **rs610913** and **rs34413328** with ***IFNA7/14* rs6475526** and ***IFNK* rs700782** were associated to an increased risk of CRC. Similarly, many genotype combinations in the shared interactions of ***CGAS* rs610913** and **rs9352000** with ***IFNA2* rs10120977, *IFNA16* rs10964912** (*r* ^2^ = .43), and ***IFNAR2* rs1131668** seemed to increase CRC risk (Table S5). Furthermore, the ***IKBKE*** SNPs, **rs15672, rs2297549, rs2297548,** the ***TBK1*** SNP **rs61933195,** and the ***TLR3*** SNP **rs3775291** showed a few interactions with the *IFN* genes (Table 5, Figure S3). There was no overlap between the *IKBKE*‐*IFNs* and *TBK1‐IFNs* interactions. Interestingly, the *IKBKE* interactions led to increased risk of CRC, while the *TLR3* interactions decreased the risk. ***IKBKE* rs2297549** shared two interactions with ***IKBKE* rs2297548,** comprising ***IFNK* rs700782** and ***IFNAR1* rs2834202,** while only one with ***IKBKE* rs15672,** with ***IFNAR1* rs2856968** (Table S5).

It is interesting to note that the interactions and r^2^ values do not seem to correlate; indeed, most of the previously genotyped *IFN* SNPs, located at the same locus on the chromosome 9 and involved in interactions with the same SNP, do not show a high LD (Figure S6).

In summary, all these regulatory SNPs could affect the expression of the corresponding genes leading to protective/harmful effects when interacting with each other.

## 4 DISCUSSION

Balance is the key to everything, especially when it concerns the immune system, which can highly contribute to both suppression and promotion of cancer. Recent studies have shown that the cGAS‐STING and TLR3 pathways, which through the TBK1‐IKKε phosphorylation induce the type I IFNs production, are disturbed in CRC, mainly because of an imbalanced expression of their coding genes.^20^, ^29^ cGAS produces cGAMP in response to cytosolic DNA, which in turn can bind and activate STING.^30^ It has been described that the levels of 2ʹ, 3ʹ ‐cGAMP, or its analogs are important for the immune system to decide which direction to follow. Indeed, high levels of STING activators have been shown to lead the immune system toward sustained inflammation and consequent tumor initiation and progression.^20^ Furthermore, cGAS plays an important role in controlling cellular senescence a delicate cellular state vital for the elimination of pre‐cancerous state but also a reservoir of potentially harmful tumorigenic progenitors.^31^ Impaired STING expression may also allow the cancer cells to escape the immunosurveillance system. Here, we showed that inherited genetic variation potentially affecting gene expression of the cGAS‐STING‐IFN pathway may contribute to CRC susceptibility. Individually, the studied SNPs showed only nominal if any associations with CRC risk, however, they seem to interact and by that affect the risk.

So far, about 100 CRC susceptibility loci have been identified through genome‐wide association studies.^5^ Polygenic risk scores derived from these studies have evaluated that some 5% of the study populations have over twofold increased risk of CRC.^32^, ^33^ In our study also, we observed an increased risk for individuals with increasing number of alleles causing a moderately increased CRC risk. However, polygenic risk scores do not take into account epistatic interactions, which may by far cause a more pronounced risk compared to single variants, as shown in our study.

In this research, the two‐way interaction, as well as the cumulative risk analyses, uncovered associations, which were substantial compared to individual SNP associations. Our results suggested that studying the interplay and/or the cumulative effects instead of the single effect of SNPs within genes involved in the immunity could be of interest to help our understanding of the mechanisms underlying the CRC development.

In our analyses, nine interactions between *CGAS, TMEM173, TBK1, and IKBKE* and further 52 interactions together with *IFNAs, IFNB, IFNW1, IFNK, IRF3, IRF7, and IFNAR1/2* in the smaller sample set were observed. For all interactions, the global null hypothesis test was highly significant (*P*‐value < .0001). Two out of the nine interactions, ***TMEM173* rs13153461*‐IKBKE* rs2297549**, and ***TMEM173* rs7380272*‐IKBKE* rs2297549**, passed the Bonferroni multiple testing corrections (*P*‐value < .0009).

The three SNPs involved in the most significant interactions were ***TMEM173* rs13153461**, which also associated with CRC risk as a single SNP, the ***TMEM173*** eQTL SNP **rs7380272**, and the ***IKBKE*** 5ʹUTR SNP **rs2297549.** Interestingly, ***TMEM173* rs13153461** and ***TMEM173* rs7380272** show a moderate LD (*r* ^2^ = .38), indicating that some of the interactions may be due to a modest LD between the SNPs.

As all the selected SNPs were potentially functional variants they are all located within enhancer/promoter histone marks, DNase hypersensitivity sites in different tissues, including gastrointestinal tract (GI) and whole blood, and are also predicted to affect several transcription factor‐binding sites (TFBSs) (Table S1). Some of them have also an eQTL nature, such as ***TMEM173* rs7380272**, whose T allele correlates with a decreased expression of *TMEM173* in blood (*P*‐values: 3.18 × 10^−31^; *Z*‐score: −11.62) (https://molgenis58.target.rug.nl/bloodeqtlbrowser/).^34^ Additionally, the selected SNPs capture many other SNPs, which can give us further information on additional SNPs or genes located at the same locus, for example ***TMEM173* rs7380272** is in LD with **rs7380824**, which is not only a missense variant mapping in a highly conserved region, predicted to be deleterious and probably damaging by SIFT and PolyPhen, respectively; it also acts as a *TMEM173* eQTL in blood tissue (*P*‐values: 2.73 × 10^−31^; *Z*‐score: −11.64) (https://molgenis58.target.rug.nl/bloodeqtlbrowser/). Hence, it could affect not only the expression of the gene, but also the function of the encoded protein.

When we included the previously genotyped *IFN* variants to our analyses, further synergistic effects became evident. The main interactions were exhibited by the four ***CGAS*** SNPs, **rs72960018, rs9352000, rs34413328,** and **rs610913,** among which a few were toward the same *IFN* SNPs**.** Particularly, **rs72960018, rs9352000,** and **rs610913** shared an interaction with the same ***IFNAR1*** SNP, **rs2856968,** which additionally interplayed with the ***IKBKE*** SNPs, **rs15672,** and **rs2297549.** A persistent increased risk was particularly exerted when their minor alleles interacted with each other. A possible explanation could be the potential involvement of *IFNAR1* rs2856968 in altering protein binding regions, as predicted by Regulome DB, such as those of FOXM1, MXI1, MAZ, MAX, and CHD1. Furthermore, it is in LD with many SNPs lying within regulatory regions, which map within TFBSs such as those of the polymerase epsilon catalytic subunit (POLE) or of AP‐2. These transcription factors (TFs) have been shown to be associated with the risk of CRC development and its progression, respectively.^35^, ^36^

On the other hand, the four ***CGAS*** SNPs were located within the binding sites of several TFs, among which NF‐κB. Aberrant regulation of NF‐κB and consequently of the downstream signaling pathways are involved in CRC initiation and progression, senescence regulation^37^, ^38^ as well as in resistance to chemotherapy and in the immune response.^39^, ^40^ Additionally, they were predicted to affect binding of several other TFs, such as Egr‐1 (early growth response‐1), YY1 (Yin Yang 1), BATF (Basic Leucine Zipper ATF‐Like Transcription Factor), that have already been reported to be associated with apoptosis and tumor cell proliferation^41^ or with tumorigenesis in CRC^42^ or to be over‐expressed in ulcerative colitis and CRC,^43^ respectively.

In this study, we included only five members of the TLR3/cGAS‐STING‐IKKε‐TBK1 signaling cascade, which has recently been reported to be disturbed in CRC due to deregulated expression of the genes involved,^19^ in addition to nine IFN genes from our previous studies to evaluate their genetic interactions. Inclusion of a large network of genes would have led to a higher number of multiple tests, increasing the likelihood of chance findings. This kind of genetic interaction study needs full genotyping data of all SNPs of interest, which lead to another limitation of our study, which is the lack of replication in another population. However, because these genes play a key role in the signaling cascade and there are emerging data about their importance in CRC, our study serves as a starting point for further studies including not only the genes and SNPs studied by us but also other genes important in the mucosal immune system.

Our data suggest that epistatic interactions and a high number of risk alleles may play an important role in explaining the CRC onset, offering novel biological understanding for the management of CRC patients. Our data warrant the exploration of these genetic variants for patient risk stratification and therapeutic decision making, including immune checkpoint inhibitors. Additionally, functional SNPs within these genes may represent potential biomarkers to be used to identify high‐CRC‐risk individuals and therefore direct them to colonoscopy. Indeed, their relative frequency within the European population (> 10%) makes them suitable for a widespread use. However, replication of these results in independent cohorts is needed, together with functional experimental studies in order to confirm the in silico‐predicted effects of the identified variants and their combinations on CRC susceptibility.

## CONFLICT OF INTEREST

The authors and planners have disclosed no potential conflicts of interest, financial or otherwise.
