# Supplementary figures and images for: Epistatic effect of TLR3 and cGAS‐STING‐IKKε‐TBK1‐IFN signaling variants on colorectal cancer risk
Source: Cancer Med. 2019 Dec 23;9(4):1473–84. doi: 10.1002/cam4.2804 (PMC7013077; doi:10.1002/cam4.2804)

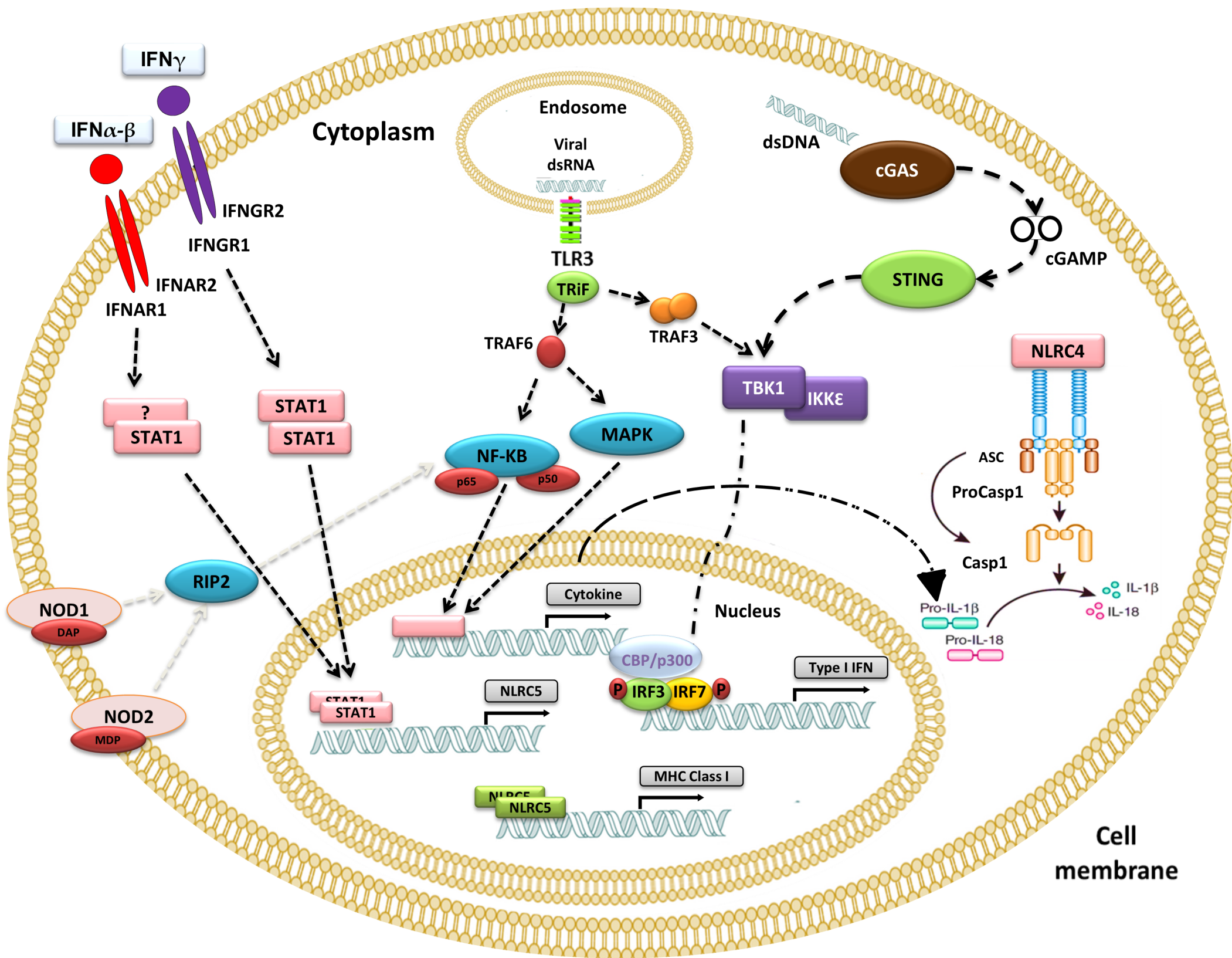

Supplement: Supplementary file 1 [file CAM4-9-1473-s001.pdf]

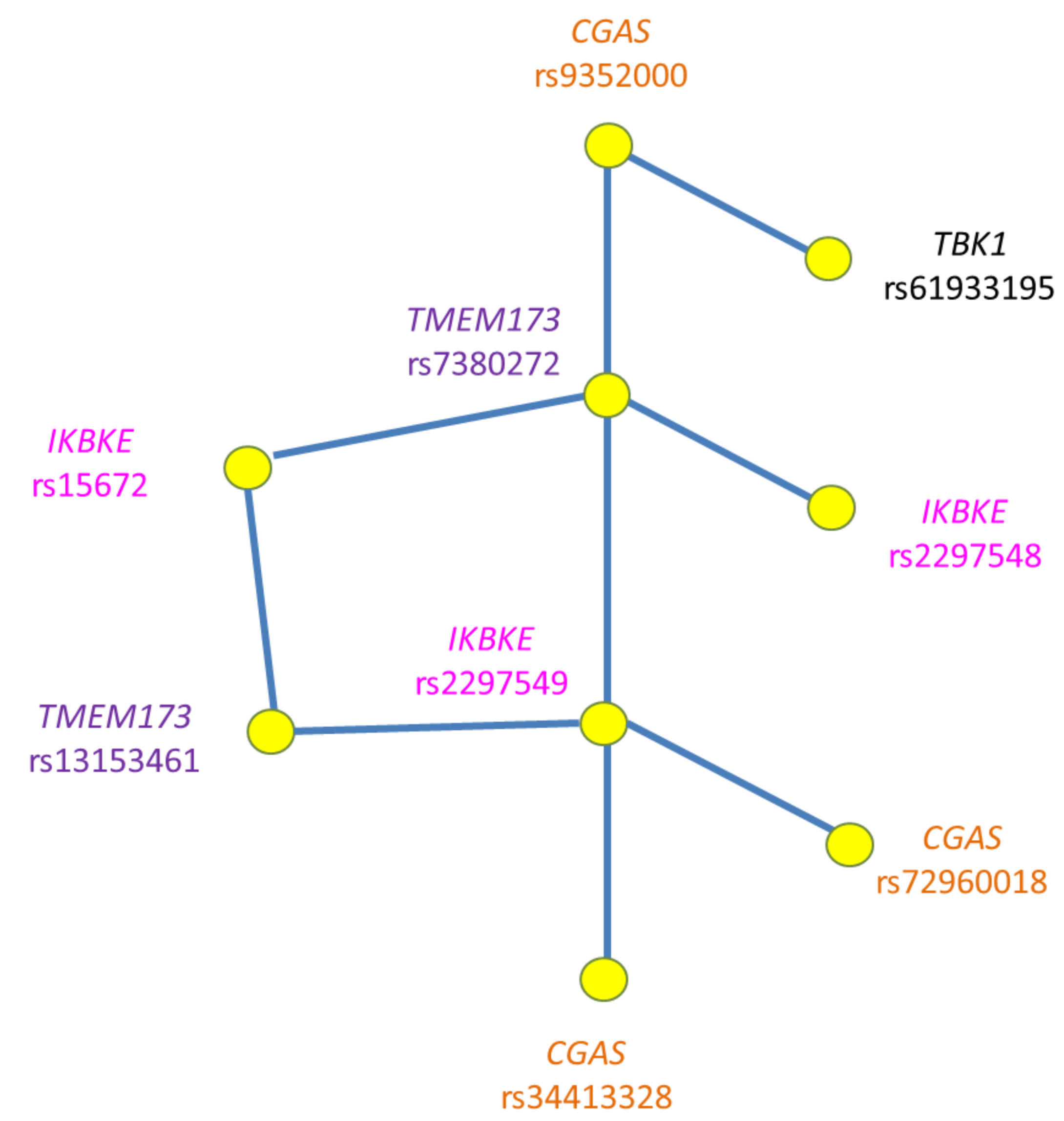

Supplement: Supplementary file 2 [file CAM4-9-1473-s002.pdf]

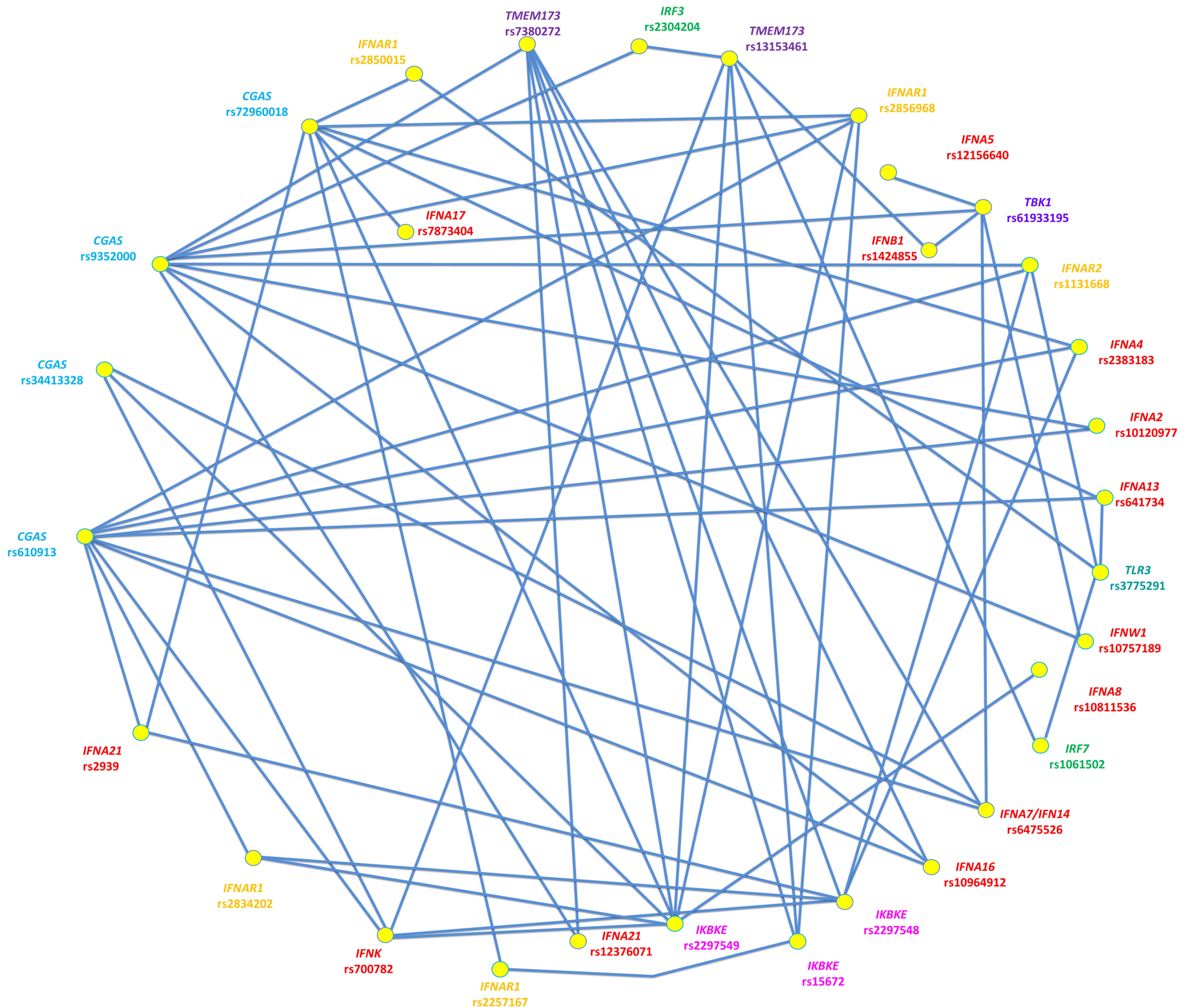

Supplement: Supplementary file 3 [file CAM4-9-1473-s003.pdf]

# TMEM173

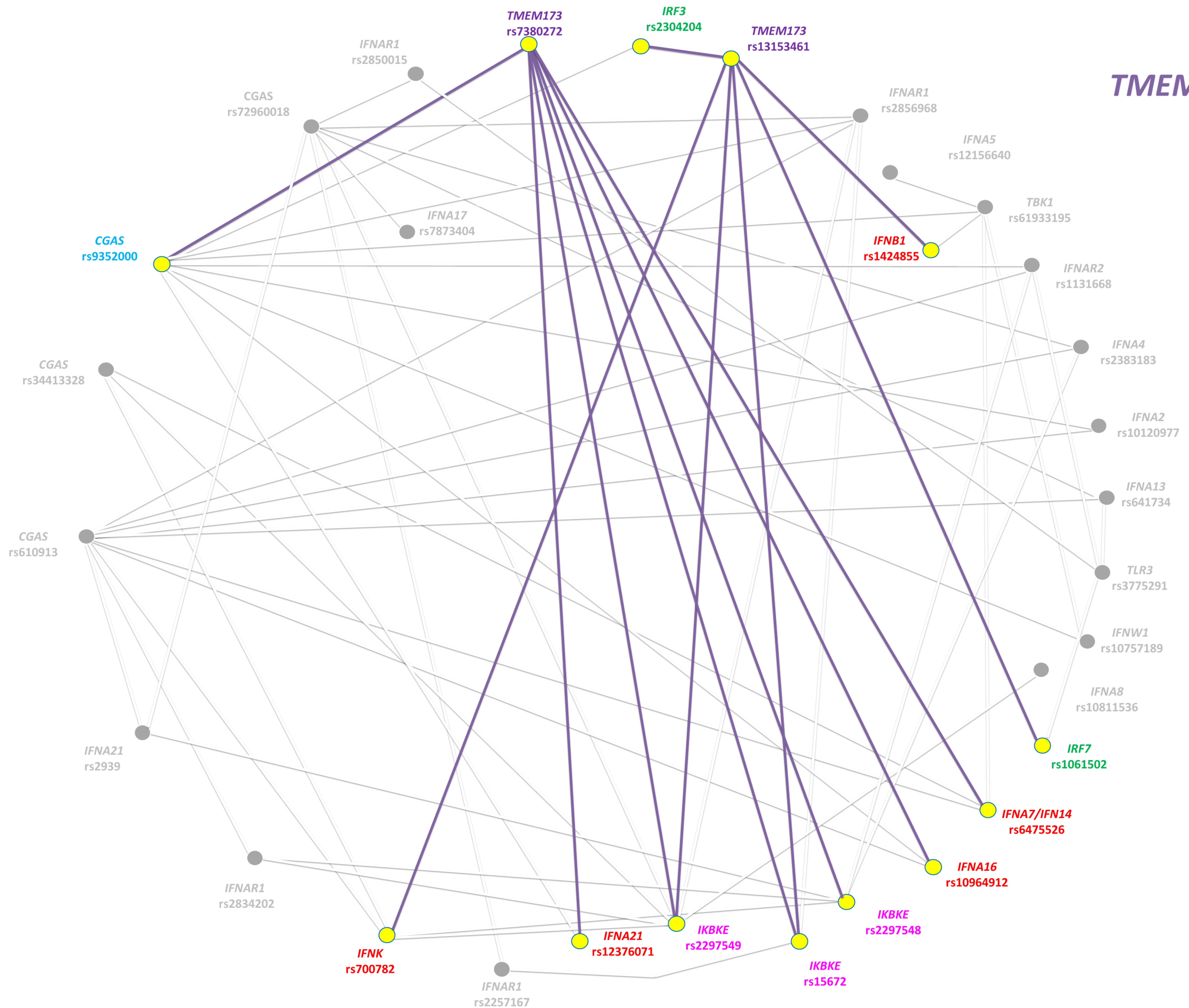

Supplement: Supplementary file 4 [file CAM4-9-1473-s004.pdf]

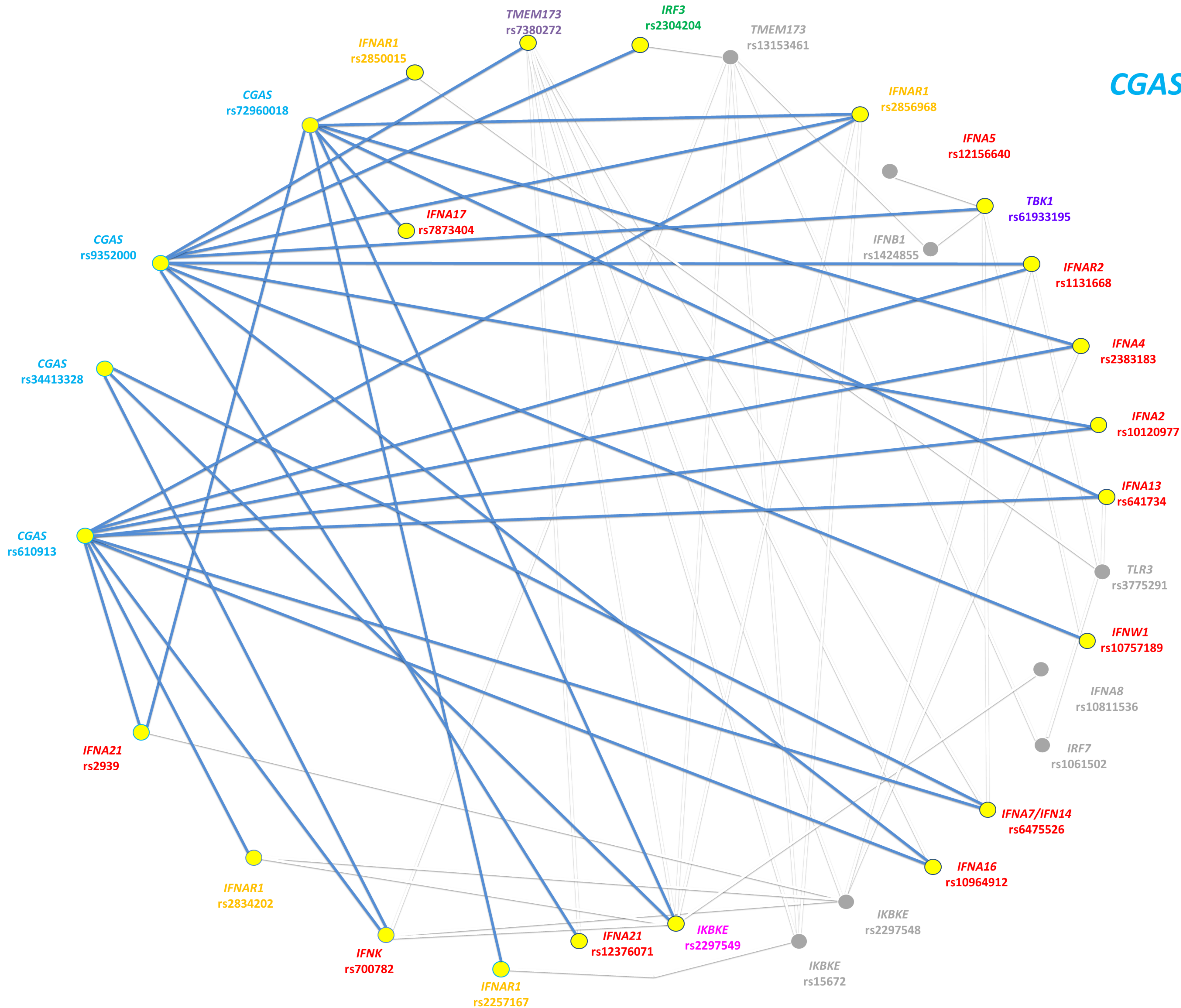

Supplement: Supplementary file 5 [file CAM4-9-1473-s005.pdf]

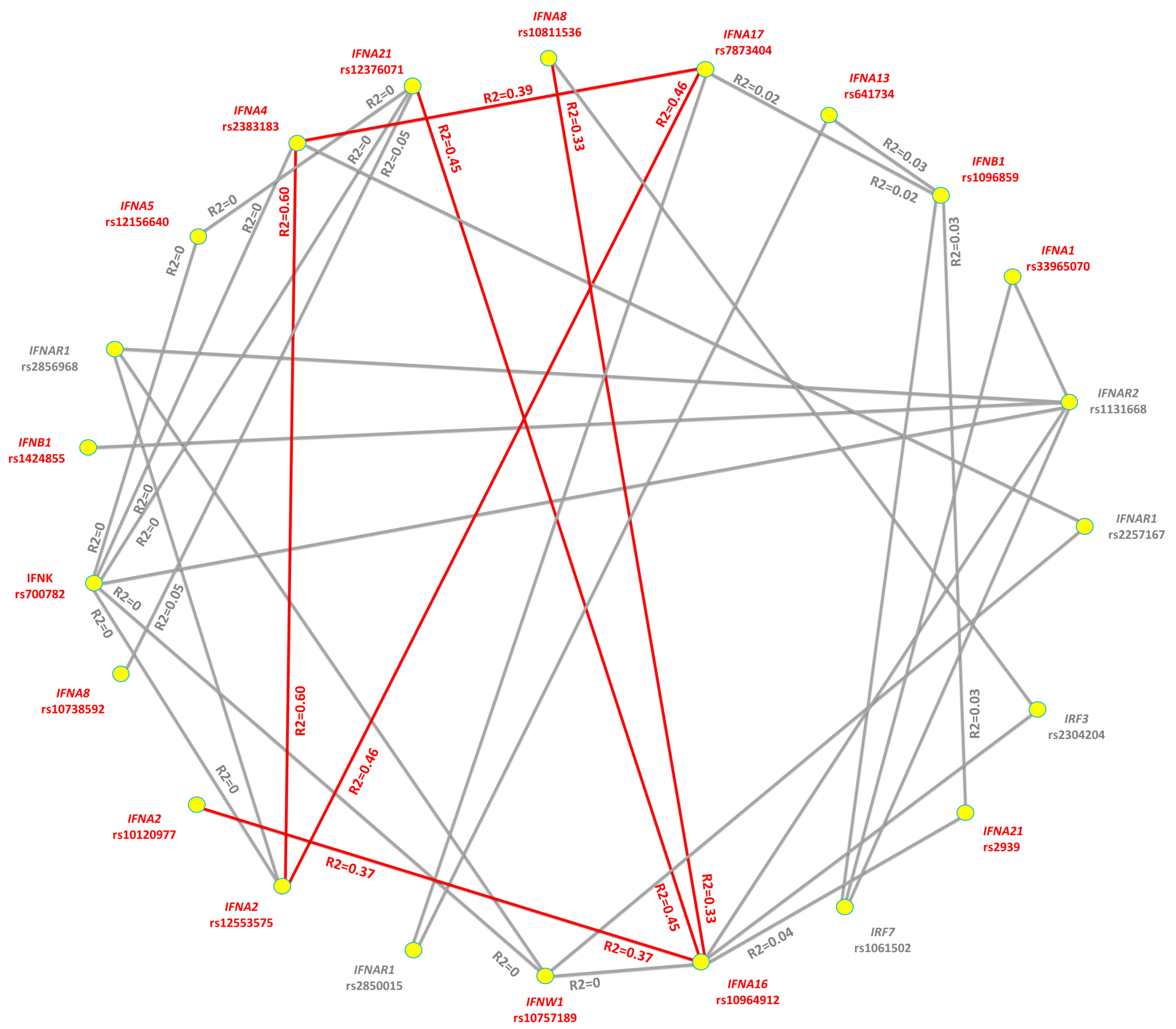

Supplement: Supplementary file 6 [file CAM4-9-1473-s006.pdf]
