# Supplementary material for: Epistatic effect of TLR3 and cGAS‐STING‐IKKε‐TBK1‐IFN signaling variants on colorectal cancer risk
Source: Cancer Med. 2019 Dec 23;9(4):1473–84. doi: 10.1002/cam4.2804 (PMC7013077; doi:10.1002/cam4.2804)
Supplement: Supplementary file 7 [file CAM4-9-1473-s007.pdf]

Table_1_SuppInfo. Bioinformatic annotation of the SNPs evaluated in *TLR3, CGAS, TMEM173, IKBKE and TBK1* genes

Haploreg							Ensembl	No Linked SNPs				MicroSNiPer		GTex		Regulome
Gene	SNP ID	Alleles	Position (hg38)	MAF (CEU)	Proteins bound	Motifs changed	Regulatory features	In BLD and/or GI	Promoter histone marks	Enhancer histone marks	Dnase	MicroRNA binding site		Effect Size/ Tissue	p-value/ Gene	Chromatin State Digestive tract/ Blood & T-cell
**TLR3**	rs3775291	TC	4: 186082920	0.32	-	AIRE	Missense	**5**	1	2	none	-	-	-	-	No data
**MB21D1**	rs610913	GT	6: 73445623	0.63	-	BATF,Bach1,GATA HMGN3	Missense	**3**	3	3	1	-	-	-	-	Strong /Weak transcription<br>Strong /Weak transcription
**MB21D1**	rs9352000	GT	6: 73452078	0.85	ELF1,E2F1,E2F4, HAE2F1,INI1,POL2	-	Missense	**4**	4	4	3		-	-	-	Active TSS<br>Active TSS/Transcr. at gene 5' and 3'/Flanking Active TSS
**MB21D1**	rs34413328	A-	6: 73452287	0.19	POL2,POL2S2,POL24 H8,NFKB,EBF1,ELF1, PU1,TBP,TCF12,TAF1 TAF7,AP2α,AP2γ, GTF2F1,STAT1,STAT3	GR, NFKB, Nkx2,PU.1, Roaz	5´ UTR variant	**6**	5	6	4	-	-	-	-	Quiescent/Low/Active TSS/Flanking Active TSS<br>Active TSS/Flanking Active TSS
**172bp 5' of MB21D1**	rs72960018	AG	6: 73452449	0.27	POL2,TBP,AP2α, AP2γ	Egr-1, HEY1, NRSF, YY1	Upstream 5´ gene variant	**1**	1	1	1	-	-	-	-	Quiescent/Low/Active TSS/Flanking Active TSS<br>Active TSS/Flanking Active TSS
**2.7kb 3' of TMEM173**	rs13153461	AG	5:139472784	0.69	-	AP4,CTCF,SMC3, TAL1	Downstream gene variant	**7**	6	7	3	-	-	0.24<br>Cells Transformed fibroblasts/ Esophagus Muscularis	1.8E-07/ TMEM173	Enhancer/Weak transcription<br>Enhancer
**TMEM173**	rs7380272	CT	5:139481019	0.15	-	AP2rep, CEBPA,CEBPB, p300	Intronic eQTL	**10**	10	10	2	-	-	-0.43<br>Cells Transformed fibroblasts/ Whole Blood/Esophagus Mucosa	3.60E-15/ TMEM173	Active TSS/Flanking Active TSS<br>Transcr. at gene 5' and 3'/Flanking Active TSS
**TBK1**	rs61933195	CA	12: 64452087	0.13	GATA2,ELF1,JUND,P U1,GATA1	Foxj1,Foxo, Pou3f2	intronic	**50**	17	31	16	-	-	-	-	Weak transcription<br>Weak transcription/Enhancer
**IKBKE**	rs2297549	AG	1:206470431	0.24	CTCF, CTCFL POL2,ELF1,POL24H8, PU1,TBP,TAF1,NFKB, PRDM1,RAD21,HEY1	AP4,E2F, TATA	5' UTR	**1**	1	1	1	-	-	-0.46<br>Esophagus Mucosa /Cells - Transformed fibroblasts	2.30E-13/ IKBKE	Active TSS/Flanking Active TSS/Enhancer<br>Active TSS/Flanking Active TSS
**IKBKE**	rs2297548	CT	1:206470715	0.81	CTCF	CTCF,ELF1,Myc, NRSF,Pou2f2, RREB1,Rad21,Sp4 YY1,ZNF219, Zfp281,Zfp740	5' UTR	**3**	3	3	2	-	-	-	-	Active TSS/Flanking Active TSS<br>Active TSS/Transcr. at gene 5' and 3'/Flanking Active TSS

**IKBKE**	rs15672	GA	1:206496625	0.49	NFKB,EBF1,PU1	Irf	3’ UTR	**33**			miR-5739 miR-920 + other 7 miRNAs	miR-6130 miR-4457 + other 13 miRNAs	0.24 Esophagus - Mucosa	7.00E-06/ IKBKE	Weak transcription/Genic Enhancers/Enhancer/Quiescent/Low Strong/Weak transcription/Genic Enhancers
								29	29	17					

Table _2_SuppInfo. Bioinformatics annotation of the interferon pathway SNPs evaluated in the previous study [1]

Haploreg							Ensembl	No Linked SNPs				MicroSNiPer		GTex		Regulome
Gene	SNP ID	Alleles	Position (hg38)	MAF (CEU)	Proteins bound	Motifs changed	Regulatory features	Total (In BLD and/or GI)	Promoter histone marks	Enhancer histone marks	Dnase	MicroRNA binding site		Effect Size/ Tissue	p-value/ Gene	Chromatin State Digestive tract/ Blood & T-cell
871bp 5' of *IFNB1*	rs1424855	C/G	9:21078815	0.38	CTCF,RAD21,ELF1	AFP1,BRCA1,FAC1,Foxj2,Foxk1,Foxo,Foxp1,Zfp105	Upstream 5´ gene variant	**7**	2	5	none	-	-	-	-	Quiescent/Low<br>Quiescent/Low/Enhancers/<br>Weak transcription/Heterochromatin
IFNB1	rs10964859	C/G	9:21140672	0.39	-	HNF1,Irf,TEF	3'-UTR	**none**	-	-	-	miR-656<br>miR-4762-5p<br>+ other 3 miRNAs	miR-4503<br>miR-3658 + other 2 miRNAs	-	-	Quiescent/Low<br>Quiescent/Low/ Weak transcription
459bp 5' of *IFNW1*	rs10757189	G/A	9:21142604	0.31	-	Nkx2,Pax-5,Pax-6	Upstream 5´ gene variant	**8**	4	7	2	-	-	GRASP gene expression of IFNB1 in dendritic cells 0.002970173793PMID:22233810		Quiescent/Low<br>Quiescent/Low/ Weak transcription
IFNA21	rs2939	T/C	9:21166004	0.13	-	HNF6,Mef2	3'-UTR	**122**	29	34	4	miR-595<br>miR-3911<br>+ other 2 miRNAs	miR-4491<br>miR-3152-3p<br>+ other 12 miRNAs	-	-	No data
242bp 5' of *IFNA21*	rs12376071	A/G	9:21166902	0.32	-	AP-1,Gfi1	Upstream 5´ gene variant	**none**	-	-	-	-	-	-	-	No data
101bp 5' of *IFNA4*	rs2383183	T/C	9:21187700	0.1	-	-	Upstream 5´ gene variant	**1**	0	1	0	-	-	-	-	Quiescent/Low<br>Quiescent/Low/ Weak Repressed PolyComb
2.6kb 3' of *IFNA16*	rs10964912	A/C	9:21218096	0.26	CTCF,YY1	LUN-1	Upstream 5´ gene variant	**1**	1	1	0	-	-	-	-	Quiescent/Low/ Weak Repressed PolyComb<br>Quiescent/Low/ Weak Repressed PolyComb
275bp 5' of *IFNA17*	rs7873404	T/C	9:21228497	0.18	-	Foxj1,Foxl1	Upstream 5´ gene variant	**86**	25	24	6	-	-	Esophagus_ Muscularis IFNA20P 6.61e-06		Quiescent/Low<br>Quiescent/Low/ Weak Repressed PolyComb
IFNA7 /IFN14	rs64755263	C/T	9:21242162	0.38	-	-	-	**none**	-	-	-	-	-	-	-	No data
928bp 5' of IFNA5	rs12156640	G/A	9:21306241	0.1	-	Bcl6b,Hoxb13,NF-kappaB,PLZF	Upstream 5´ gene variant	1	none	1	none	-	-	-	-	Quiescent/Low/ Weak Repressed PolyComb<br>Quiescent/Low/ Weak Repressed PolyComb
851bp 5' of IFNA13	rs641734	A/T	9:21368927	0.18	-	-	Upstream 5´ gene variant	**58**	40	16	7	-	-	-	-	No data

IFNA2	rs10120977	A/G	9:21384363	0.25	-	HMG-IY,TATA	3'-UTR	**3**	1	2	1	miR-6128 miR-890 + other 11 miRNAs	miR-1282 miR-2681-5p	-	-	Quiescent/Low/ Weak Repressed PolyComb<br><br>Quiescent/Low/ Weak Transcription/Weak Repressed PolyComb/ Repressed PolyComb
647bp 5' of IFNA8	rs12553575	A/G	9:21408498	0.16	-	AFP1,Cdx2,Dbx1,Hlx1,Hoxa10,Hoxa9,Hoxb8,Hoxc9,Hoxd10,Lhx3,Nkx2,Nkx3,Pou3f4,Sox	Upstream 5´ gene variant	**5**	4	5	none	-	-	-	-	Quiescent/Low/ Weak Repressed PolyComb<br><br>Quiescent/Low/Weak Repressed PolyComb/ Repressed PolyComb
629bp 5' of *IFNA8*	rs10738592	C/T	9:21408516	0.48	-	Foxa,Ik-2,Ncx,STAT,Sox	Upstream 5´ gene variant	**5**	4	2	1	-	-	-	-	No data
452bp 5' of *IFNA8*	rs10811536	T/C	9:21408693	0.23	-	CTCF,Fox,Foxp1, Pou1f1,TATA	Upstream 5´ gene variant	**3**	2	2	1	-	-	-	-	Quiescent/Low/ Weak Repressed PolyComb<br><br>Quiescent/Low/Weak Repressed PolyComb/ Repressed PolyComb
IFNA1	rs33965070	C/G	9:21440994	0.18	-	HES1	missense	**none**	-			-	-	-	-	Quiescent/Low/ Weak Repressed PolyComb<br>Quiescent/Low/ Weak Repressed PolyComb
IFNK	rs700782	G/A	9:27526047	0.24	-	Nanog,Pou2f2, Pou5f1	3'-UTR	**75**	22	45	13	miR-33b-5p miR-450a-5p	miR-153 miR-450a-5p + other 4 miRNAs	-	-	Quiescent/Low/Enhancers/Weak transcription<br>Quiescent/Low/ Weak Repressed PolyComb/Weak Transcription
IRF3	rs2304204	A/G	19:50169020	0.22	POL2,SRF,TBP,USF2,SP1,ELK4,POL2S2,CTCF,ETS1,IRF1,YY1,GATA1	Nanog	5'-UTR	**14**	14	14	7	-	-	Whole_Blood IRF3 4.8 E-106		Active TSS<br><br>Active TSS
IRF7	rs1061502	A/G	11:614318	0.28	AP-4,CTCF,E4F1, MZF1::1-4,Myf	-	missense	**27**	25	25	19	-	-	-	-	Transcr. at gene 5' and 3'/ Genic Enhancers/Enhancer/ Flanking Active TSS/ Active TSS<br><br>Transcr. at gene 5' and 3'/ Genic Enhancers/Enhancer/ Flanking Active TSS/ Strong Transcription
IFNAR1	rs2856968	A/G	21:33325676	0.42	TBP,HAE2F1	-	intronic	**22**	11	22	10	-	-	Whole_Blood IL10RB 1.21E-11		Flanking Active TSS/ Active TSS<br>Flanking Active TSS/ Active TSS

IFNAR1	rs2850015	C/T	21:34697264	0.31	POL2,NRF1,POL24H8,YY1,TAF1,TBP,TCF4,MAX,CMYC,MXI1,FOSL2,SIN3AK20,CCNT2,CJUN,E2F6,ELF1,HEY1,HMGN3,IRF1,E2F4,HAE2F1,USF1	Ahr::Arnt::HIF1,E2F,Egr1,HDAC2,HIF1,Mtf1,Nrf1,Pax-5,SETDB1,Sp4	5'-UTR	**8**			-	-	Cells Transformed fibroblasts IFNAR1 2.16e-07	Esophagus_ Mucosa IFNAR1 1.4 e-08 Esophagus_ Muscularis IFNAR1 2.23e-06	Active TSS<br>Active TSS/ Transcr. at gene 5' and 3'
								7	8	5					
IFNAR1	rs2257167	G/C	21:34715699	0.13	-	BCL,GATA,HDAC2	missense	**1**			-	-	Whole_Blood IFNAR1 4.18E-73		No data
								1	1	none					
IFNAR1	rs2834202	A/G	21:34730954	0.26	-	HNF4,RAR	3'-UTR	**9**			miR-302a-5p	miR-513b miR-5003-5p + other 2 miRNAs	Whole_Blood IFNAR1 4.87E-49	Whole_Blood IFNAR2 0.0013	Weak Transcription<br>Weak Transcription
								5	9	2					
IFNAR2	rs1131668	G/A	21: 33262573	0.33	-	-	missense	**27**			-	-	Whole_Blood IFNAR2 1.75e-13	IFNAR2 Esophagus Mucosa(3.24 e-23) Muscolaris (5.15e-07) and colon transverse (1.27e-12)	No data
								23	27	16					

[1] Lu, S. (2014) Single nucleotide polymorphisms within interferon signaling pathway genes are associated with colorectal cancer susceptibility and survival. PLoS One vol. 9, pp. e111061.

Table_3_SuppInfo. False-Positive Report Probability Values for Associations Between the Risk of Colorectal Cancer and the frequency of genotypes/alleles that showed to be associated

SNP ID	Genotype	OR	95%CI	P-value	Statistical Power	Prior Probability				
						**0.25**	**0.1**	**0.01**	**0.001**	**0.0001**
**rs72960018**	A/G vs. AA	1.6	(1.04-2.46)	**0.03**	0.38	0.23	0.46	0.88	0.99	1
	G/G vs. AA	1.73	(1.13-2.63)	**0.01**	0.25	**0.13**	0.29	0.79	0.98	1
	A/G+G/G vs. AA	1.68	(1.11-2.53)	**0.01**	0.29	**0.14**	0.31	0.80	0.98	1
**rs9352000**	G/G vs. T/T	2.04	(1.07-3.88)	**0.03**	0.17	0.38	0.63	0.94	0.99	1
	G/G vs. T/T+G/T	2.02	(1.07-3.84)	**0.03**	0.18	0.39	0.64	0.94	0.99	1
**rs13153461**	G/G vs. A/A	1.6	(1.07-2.39)	**0.02**	0.38	**0.17**	0.37	0.84	0.98	1
	G/G vs. A/A+A/G	1.53	(1.03-2.27)	**0.03**	0.46	0.21	0.43	0.87	0.99	1
**3-4 alleles**	-	1.31	(1.06-1.62)	**0.01**	0.89	**0.05**	**0.12**	0.56	0.93	0.99
**5-6 alleles**	-	2.98	(1.35- 6.56)	**0.007**	0.04	0.35	0.60	0.93	0.99	1

CI, confidence interval; OR, odds ratio;
Statistical power was calculated using the number of observations in the subgroup and the OR and P values in this table.

Table_4_SuppInfo. SNP-SNP pair-wise interactions for *TLR3, cGAS, TMEM173, TBK1* and *IKBKE* . Age and sex adjusted ORs and 95% CI were calculated for the best model of each pair with the overal p-value based on the LRT

***TMEM173*<br>rs7380272**	***IKBKE*<br>rs2297549**<br>TT+TC	CC
0	1.00	2.37<br>(1.30-4.32)<br>**0.005**
1	1.38<br>(1.10-1.72)<br>**0.0051**	0.70<br>(0.35-1.38)<br>0.30
2	1.90 (1.21-2.97)<br>**0.0051**	0.21 (0.05-0.81)<br>**0.02**
		**0.0004**

***TMEM173*<br>rs13153461**	***IKBKE*<br>rs2297549**<br>TT+TC	CC
AA+AG	1.00	1.84 (1.08-3.14)<br>**0.025**
GG	1.84<br>(1.21-2.82)<br>**0.0046**	0.31 (0.088-1.07)<br>0.06
		**0.0007**

***TMEM17 3*<br>rs1315346 1**	***IKBKE*<br>rs15672**<br>0	1	2
0	1.00	0.84<br>(0.70-1.00)<br>0.06	0.71<br>(0.50-1.01)<br>0.06
1	0.94<br>(0.72-1.22)<br>0.63	1.01<br>(0.81-1.28)<br>0.90	1.10<br>(0.79-1.52)<br>0.58
2	0.88<br>(0.52-1.49)<br>0.64	1.22<br>(0.85-1.75)<br>0.27	1.70<br>(1.00-2.89)<br>**0.05**
			**0.031**

***TMEM173*<br>rs7380272**	***IKBKE*<br>rs15672**<br>GG+GA	AA
CC	1.00	0.70<br>(0.53-0.92)<br>**0.01**
CT+TT	1.03<br>(0.78-1.36)<br>0.83	1.40<br>(0.84-2.32)<br>0.2
		**0.022**

***MB21D1*<br>rs72960018**	***IKBKE*<br>rs2297549**<br>TT	TC+CC
GG+GA	1.00	1.07 (0.87-1.31)<br>0.55
AA	1.01<br>(0.57-1.76)<br>0.98	0.37<br>(0.19-0.71)<br>**0.0031**
		**0.018**

***MB21D1*<br>rs9352000**	***TMEM173*<br>rs7380272**<br>0	1	2
TT+TG	1.00	1.15<br>(0.92-1.43)<br>0.22	1.32<br>(0.85-2.05)<br>0.22
GG	1.43<br>(0.70-2.90)<br>0.32	15.19<br>(1.72-134.3)<br>**0.014**	161.5<br>(1.97-13235.6)<br>**0.024**
			**0.004**

***TMEM173*<br>rs7380272**	**IKBKE<br>rs2297548**<br>TT+TC	CC
CC	1.00	0.92<br>(0.52-1.63)<br>0.78
CT+TT	1.14<br>(0.89-1.46)<br>0.29	7.47<br>(1.75-31.86)<br>**0.0066**
		**0.015**

***MB21D1*<br>rs9352000**	***TBK1*<br>rs61933195**<br>CC	CA+AA
TT+TG	1.00	0.83<br>(0.66-1.05)<br>0.12
GG	1.49<br>(0.69-3.22)<br>0.31	6.41<br>(1.50-27.43)<br>**0.0123**
		**0.008**

***MB21D1*<br>rs3441332 8**	***IKBKE*<br>rs2297549**<br>TT	TC	CC
AA	1.00	0.76<br>(0.59-0.99)<br>**0.045**	1.74<br>(0.93-3.25)<br>0.08
A-+--	0.81<br>(0.63-1.06)<br>0.12	1.19<br>(0.87-1.64)<br>0.28	0.72<br>(0.33-1.58)<br>0.41
			**0.016**

Table_5_SuppInfo . SNP-SNP pair-wise interactions for *TLR3, cGAS, TMEM173, TBK1, IKBKE* and *IFN* genes.

Age and sex adjusted ORs and 95% CI were calculated for the best model of each pair with the overal p-value based on the LRT

**IRF3<br>rs2304204**	**TMEM173<br>rs13153461**	
	AA+AG	GG
AA	1.00	1.05<br>(0.49-2.24)<br>0.90
AG	1.11<br>(0.85-1.44)<br>0.45	4.02<br>(1.83-8.87)<br>**0.001**
GG	0.84<br>(0.53-1.33)<br>0.46	0.22<br>(0.02-2.07)<br>0.18
		**0.004**

**TMEM173<br>rs13153461**	**IRF7<br>rs1061502**	
	TT	TC+CC
AA	1.00	0.87<br>(0.62-1.21)<br>0.41
AG	0.76<br>(0.54-1.07)<br>0.12	1.38<br>(0.92-2.05)<br>0.12
GG	1.58<br>(0.79-3.16)<br>0.20	1.53<br>(0.67-3.48)<br>0.31
		**0.042**

**IFNB1<br>rs1424855**	**TMEM173<br>rs13153461**	
	AA+AG	GG
CC	1.00	2.95<br>(1.29-6.72)<br>**0.01**
CG	1.06<br>(0.81-1.40)<br>0.65	0.87<br>(0.41-1.86)<br>0.71
GG	0.97<br>(0.64-1.45)<br>0.87	4.82<br>(1.03-22.61)<br>**0.05**
		**0.035**

**IFNK<br>rs700782**	**TMEM173<br>rs13153461**	
	AA+AG	GG
GG	1.00	1.14<br>(0.60-2.17)<br>0.70
GA+AA	1.22<br>(0.94-1.58)<br>0.13	4.16<br>(1.73-9.97)<br>**0.001**
		**0.006**

**TMEM173<br>rs7380272**	**IFNA7/14<br>rs6475526**		
	0	1	2
0	1.00	1.33<br>(1.08-1.63)<br>**0.006**	1.76<br>(1.17-2.65)<br>**0.006**
1	1.57<br>(1.04-2.36)<br>**0.03**	1.39<br>(1.01-1.91)<br>**0.05**	1.23<br>(0.72-2.09)<br>0.46
2	2.47<br>(1.09-5.57)<br>**0.03**	1.45<br>(0.82-2.56)<br>0.20	0.85<br>(0.31-2.32)<br>0.75
			**0.025**

**TMEM173<br>rs7380272**	**IFNA16<br>rs10964912**	
	AA	AC+CC
CC+CT	1.00	1.31<br>(1.02-1.70)<br>**0.04**
TT	3.05<br>(0.86-10.82)<br>0.09	0.36<br>(0.08-1.66)<br>0.19
		**0.029**

**TMEM173<br>rs7380272**	**IFNA21<br>rs12376071**		
	0	1	2
CC+CT	1.00	1.18<br>(0.98-1.43)<br>0.09	1.40<br>(0.95-2.06)<br>0.09
TT	5.28<br>(1.22-<br>22.86)<br>**0.02**	0.72<br>(0.26-2.02)<br>0.53	0.10<br>(0.01-0.89)<br>**0.04**
			**0.022**

**MB21D1<br>rs72960018**	**IFNA4<br>rs2383183**	
	TT	TC+CC
0	1.00	1.26<br>(0.85-1.87)<br>0.25
1	0.91<br>(0.72-1.15)<br>0.43	0.60<br>(0.40-0.90)<br>**0.01**
2	0.83<br>(0.52-1.32)<br>0.43	0.29<br>(0.13-0.61)<br>**0.001**
		**0.009**

**MB21D1<br>rs610913**	**IFNA4<br>rs2383183**	
	TT	TC+CC
TT+TG	1.00	0.70<br>(0.50-0.99)<br>**0.04**
GG	1.08<br>(0.73-1.58)<br>0.71	0.70<br>(0.50-0.99)<br>**0.04**
		**0.03**

**MB21D1<br>rs72960018**	**IFNA13<br>rs641734**	
	CC	CT+TT
GG	1.00	1.12<br>(0.79-1.60)<br>0.53
GA	1.07<br>(0.77-1.48)<br>0.68	0.57<br>(0.38-0.84)<br>**0.01**
AA	0.54<br>(0.29-1.03)<br>0.06	0.67<br>(0.25-1.80)<br>0.42
		**0.012**

**IFNA13<br>rs641734**	**MB21D1<br>rs610913**		
	0	1	2
CC	1.00	0.98<br>(0.78-1.22)<br>0.83	0.95<br>(0.61-1.49)<br>0.83
CT+TT	0.57<br>(0.38-0.86)<br>**0.007**	0.82<br>(0.59-1.13)<br>0.23	1.17<br>(0.72-1.90)<br>0.53
			**0.035**

**IFNK<br>rs700782**	**MB21D1<br>rs34413328**	
	AA	A-+-/-
GG	1.00	1.35<br>(0.97-1.86)<br>0.07
GA+AA	1.66<br>(1.20-2.30)<br>**0.002**	1.14<br>(0.79-1.66)<br>0.48
		**0.016**

**IFNK rs700782**	**MB21D1 rs610913**		
	0	1	2
GG	1.00	1.30<br>(1.03-1.64)<br>**0.03**	1.68<br>(1.05-2.68)<br>**0.03**
GA+AA	1.90<br>(1.28-2.81)<br>**0.001**	1.63<br>(1.18-2.24)<br>**0.003**	1.39<br>(0.88-2.21)<br>0.16

0.01

**IFNA7/14 rs6475526**	**MB21D1 rs34413328**		
	0	1	2
CC	1.00	1.38<br>(0.99-1.92)<br>0.06	1.90<br>(0.99-3.68)<br>0.06
CT+TT	1.63<br>(1.19-2.24)<br>**0.0025**	1.41<br>(1.01-1.96)<br>**0.04**	1.22<br>(0.72-2.06)<br>0.47

0.023

**IFNA7/14 rs6475526**	**MB21D1 rs610913**		
	0	1	2
0	1.00	1.37<br>(1.05-1.78)<br>**0.02**	1.87<br>(1.11-3.16)<br>**0.02**
1	1.52<br>(1.14-2.03)<br>**0.004**	1.58<br>(1.19-2.11)<br>**0.002**	1.65<br>(1.10-2.47)<br>**0.01**
2	2.31<br>(1.30-4.10)<br>**0.004**	1.83<br>(1.21-2.77)<br>**0.004**	1.46<br>(0.83-2.54)<br>0.19

0.016

**MB21D1 rs9352000**	**IFNA2 rs10120977**	
	AA	AG+GG
TT+TG	1.00	1.34<br>(1.03-1.76)<br>**0.03**
GG	3.86<br>(1.33-11.21)<br>**0.013**	0.93<br>(0.26-3.29)<br>0.9

0.016

**MB21D1 rs610913**	**IFNA2 rs10120977**	
	AA	AG+GG
TT+TG	1.00	1.35<br>(1.01-1.79)<br>**0.04**
GG	1.78<br>(1.14-2.79)<br>**0.01**	1.04<br>(0.63-1.71)<br>0.88

0.03

**MB21D1 rs9352000**	**IFNA16 rs10964912**	
	AA	AC+CC
TT+TG	1.00	1.36<br>(1.04-1.78)<br>**0.02**
GG	10.54<br>(2.31-48.2)<br>**0.002**	1.48<br>(0.53-4.13)<br>0.45

0.002

**MB21D1 rs610913**	**IFNA16 rs10964912**	
	AA	AC+CC
TT+TG	1.00	1.41<br>(1.06-1.87)<br>**0.02**
GG	1.83<br>(1.15-2.93)<br>**0.01**	1.25<br>(0.77-2.02)<br>0.37

0.02

**MB21D1 rs9352000**	**IFNAR2 rs1131668**	
	GG	GA+AA
TT+TG	1.00	0.99<br>(0.77-1.27)<br>0.96
GG	12.75<br>(1.50-109.4)<br>**0.02**	1.03<br>(0.38-2.80)<br>0.95

0.027

**MB21D1 rs610913**	**IFNAR2 rs1131668**		
	0	1	2
TT+TG	1.00	1.07<br>(0.87-1.31)<br>0.55	1.13<br>(0.75-1.71)<br>0.55
GG	2.06<br>(1.24-3.42)<br>**0.006**	1.15<br>(0.79-1.68)<br>0.47	0.64<br>(0.33-1.27)<br>0.20

0.037

**IKBKE rs2297548**	**IFNK rs700782**		
	GG	GA	AA
TT	1.00	1.12<br>(0.82-1.55)<br>0.47	1.42<br>(0.69-2.89)<br>0.34
TC+CC	1.04<br>(0.74-1.45)<br>0.83	1.87<br>(1.24-2.82)<br>**0.003**	0.50<br>(0.18-1.37)<br>0.18

0.028

**IKBKE rs2297549**	**IFNK rs700782**		
	GG	GA	AA
CC	1.00	1.31<br>(0.94-1.83)<br>0.11	0.58<br>(0.27-1.24)<br>0.16
CT+TT	0.89<br>(0.64-1.23)<br>0.48	1.21<br>(0.82-1.78)<br>0.34	2.50<br>(0.93-6.72)<br>0.07

0.05

**IKBKE rs2297548**	**IFNAR1 rs2834202**	
	AA	AG+GG
TT+TC	1.00	1.31<br>(1.01-1.70)<br>**0.04**
CC	3.82<br>(1.62-9.01)<br>**0.002**	1.15<br>(0.37-3.58)<br>0.81

0.004

**IKBKE rs2297549**	**IFNAR1 rs2834202**		
	0	1	2
CC	1.00	0.95 (0.73-1.22) 0.67	0.89 (0.54-1.49) 0.67
CT+TT	0.77 (0.56-1.06) 0.11	1.32 (0.92-1.89) 0.13	2.25(1.18-4.28) **0.014**

0.031

**IKBKE rs15672**	**IFNAR1 rs2856968**	
	AA	AG+GG
GG	1.00	2.47 (1.54-3.98) **0.0002**
GA+AA	1.72 (1.13-2.62) **0.01**	1.85 (1.24-2.76) **0.003**

0.002

**IKBKE rs2297549**	**IFNAR1 rs2856968**		
	AA	AG	GG
TT	1.00	1.20 (0.85-1.69) 0.31	1.01 (0.62-1.62) 0.98
TC	0.75 (0.50-1.13) 0.17	1.16 (0.77-1.74) 0.47	2.24 (1.16-4.31) **0.02**
CC	0.78 (0.33-1.86) 0.58	6.30 (1.71-23.18) **0.006**	0.56 (0.08-3.86) 0.56

0.004

**TKB1 rs61933195**	**IFN5 rs12156640**	
	GG	GA+AA
CC	1.00	1.39 (0.97-1.98) 0.07
CA+AA	1.30 (0.94-1.81) 0.11	0.55 (0.33-0.93) **0.027**

0.008

**MB21D1 rs9352000**	**IRF3 rs2304204**	
	AA	AG+GG
TT+TG	1.00	1.14 (0.88-1.47) 0.33
GG	0.93 (0.34-2.56) 0.89	9.42 (2.14-41.53)

0.01

**TLR3 rs3775291**	**IFNAR2 rs1131668**		
	0	1	2
CC+CT	1.00	0.85 (0.70-1.03) 0.10	0.72 (0.49-1.06) 0.10
TT	0.38 (0.21-0.68) **0.0012**	0.94 (0.57-1.56) 0.82	2.36 (0.91-6.08) 0.08

0.004

**IKBKE rs15672**	**IFNAR1 rs2257167**	
	GG	GC+CC
0	1.00	2.19 (1.37-3.52) **0.001**
1	1.14 (0.93-1.4) 0.21	1.35 (0.96-1.89) 0.08
2	1.30 (0.86-1.96) 0.21	0.83 (0.52-1.33) 0.44

0.008

**IFNA17 rs7873404**	**MB21D1 rs72960018**	
	GG	GA+AA
TT	1.00	0.99 (0.71-1.37) 0.95
TC	1.41 (0.99-2.01) 0.06	0.69 (0.48-1.00) **0.05**
CC	0.56 (0.25-1.25) 0.16	0.87 (0.35-2.17) 0.76

0.019

**MB21D1 rs610913**	**IFNA21 rs2939**	
	TT	TC+TT
0	1.00	0.55 (0.37-0.82) **0.003**
1	0.92 (0.74-1.15) 0.48	0.79 (0.57-1.09) 0.15
2	0.85 (0.54-1.33) 0.48	1.13 (0.70-1.84) 0.61

0.023

**TLR3 rs3775291**	**IFNAR1 rs2850015**		
	0	1	2
CC+CT	1.00	0.97 (0.79-1.18) 0.74	0.93 (0.62-1.40) 0.74
TT	1.23 (0.67-2.26) 0.51	0.54 (0.32-0.91) **0.02**	0.24 (0.09-0.66) **0.006**

0.04

**TKB1 rs61933195**	**IFNB1 rs1424855**		
	0	1	2
CC	1.00	0.84 (0.67-1.03) 0.10	0.70 (0.46-1.07) 0.10
CA+AA	0.64 (0.42-0.96) **0.03**	1.00 (0.71-1.40) 1.00	1.57 (0.89-2.77) 0.12

0.031

**IKBKE rs2297548**	**IFNA21 rs2939**	
	TT	TC+TT
0	1.00	1.01 (0.74-1.37) 0.97
1	1.45 (1.10-1.91) **0.008**	0.87 (0.61-1.23) 0.43
2	2.11 (1.21-3.66) **0.008**	0.75 (0.39-1.43) 0.38

0.02

MB21D1 rs72960018	IFNAR1 rs2856968		
	0	1	2
GG	1.00	1.01 (0.80-1.28) 0.94	1.02 (0.63-1.65) 0.94
GA+AA	0.55 (0.38-0.80) **0.0015**	0.94 (0.68-1.29) 0.69	1.59 (0.99-2.55) **0.05**

0.001

TKB1 rs61933195	IFNA7/14 rs6475526	
	CC	CT+TT
0	1.00	1.62 (1.21-2.16) **0.001**
1	1.42 (0.96-2.09) 0.08	1.28 (0.89-1.85) 0.18
2	2.00 (0.91-4.38) 0.08	1.02 (0.54-1.93) 0.95

0.011

MB21D1 rs610913	IFNAR1 rs2834202		
	AA	AG	GG
TT+TG	1.00	1.46 (1.08-1.97) **0.01**	0.91 (0.51-1.61) 0.74
GG	1.62 (1.03-2.53) **0.035**	1.04 (0.62-1.75) 0.88	8.13 (1.37-48.14) **0.02**

0.009

IKBKE rs2297549	IFNA8 rs10811536		
	TT	TC	CC
TT+TC	1.00	1.36 (1.03-1.79) **0.03**	1.25 (0.67-2.36) 0.48
CC	2.00 (0.85-4.72) 0.11	1.43 (0.54-3.79) 0.47	0.04 (0.003-0.65) **0.02**

0.033

TKB1 rs61933195	IFNW1 rs10757189	
	GG	GA+AA
CC	1.00	1.40 (1.05-1.87) **0.02**
CA+CC	1.49 (1.00-2.21) **0.05**	0.94 (0.63-1.40) 0.76

0.033

MB21D1 rs72960018	IFNA21 rs2939_CT	
	TT+TC	CC
GG+GA	1.00	1.19 (0.84-1.69) 0.33
AA	1.02 (0.75-1.39) 0.91	0.59 (0.41-0.84) **0.004**

0.005

MB21D1 rs9352000	IFNAR1 rs2856968	
	AA+AG	GG
TT+TG	1.00	1.01 (0.70-1.47) 0.96
GG	1.29 (0.51-3.28) 0.59	9.57 (1.77-51.80) **0.009**

0.035

MB21D1 rs610913	IFNAR1 rs2856968		
	AA	AG	GG
TT+TG	1.00	1.58 (1.17-2.14) **0.003**	1.37 (0.91-2.06) 0.13
GG	1.88 (1.10-3.20) **0.02**	1.30 (0.79-2.14) 0.30	6.44 (2.12-19.53) **0.001**

0.001

IKBKE rs2297548	IFN44 rs2383183	
	TT	TC+CC
0	1.00	1.08 (0.75-1.55) 0.67
1	1.38 (1.07-1.78) **0.01**	0.84 (0.55-1.30) 0.44
2	1.90 (1.14-3.15) **0.01**	0.66 (0.28-1.51) 0.32

0.045

MB21D1 rs72960018	IFNAR1 rs2850015	
	CC	CT+TT
GG	1.00	1.05 (0.75-1.47) 0.77
AG	1.13 (0.78-1.64) 0.53	0.66 (0.45-0.97) **0.03**
AA	0.49 (0.23-1.03) 0.06	0.88 (0.39-1.97) 0.75

0.045

TLR3 rs3775291	IRF7 rs1061502	
	TT+TC	CC
CC	1.00	0.48 (0.23-1.01) **0.05**
CT	0.75 (0.57-0.99) **0.04**	1.36 (0.66-2.84) 0.41
TT	0.72 (0.45-1.14) 0.16	0.09 (0.01-0.78) **0.03**

0.016

MB21D1 rs9352000	IFNA21 rs12376071	
	AA	AG+GG
TT+TG	1.00	1.26 (0.97-1.63) 0.08
GG	10.77 (2.36-49.18) **0.002**	1.30 (0.49-3.43) 0.60

0.005

MB21D1 rs9352000	IFNW1 rs10757189	
	GG+GA	AA
TT+TG	1.00	1.01 (0.60-1.71) 0.97
GG	3.31 (1.38-7.93) **0.007**	0.08 (0.01-0.75) **0.03**

0.009

IKBKE rs2297548	IFNAR2 rs1131668	
	GG+GA	AA
TT+TC	1.00	1.07 (0.72-1.61) 0.72
CC	2.17 (1.08-4.35) **0.03**	0.04 (0.001-1.84) 0.10

0.026

IFNA13 rs641734	TLR3 rs3775291	
	CC	CT+TT
CC	1.00	0.97 (0.71-1.31) 0.83
CT+TT	1.10 (0.75-1.62) 0.62	0.63 (0.43-0.90) **0.01**

0.035

MB21D1 rs72960018	IFNAR1 rs2257167		
	0	1	2
GG	1.00	0.91 (0.65-1.26) 0.56	10.82 (0.43-1.58) 0.56
GA+AA	0.65 (0.49-0.88) **0.005**	1.14 (0.79-1.67) 0.48	2.00 (1.00-4.02) **0.05**

0.008
