# Supplementary material for: Epistatic effect of TLR3 and cGAS‐STING‐IKKε‐TBK1‐IFN signaling variants on colorectal cancer risk
Source: Cancer Med. 2019 Dec 23;9(4):1473–84. doi: 10.1002/cam4.2804 (PMC7013077; doi:10.1002/cam4.2804)
Supplement: Supplementary file 8 [file CAM4-9-1473-s008.docx]

**SUPPLEMENTARY FIGURES LEGENDS**

**Figure _1_SuppInfo.pdf** A protein interaction network representing how the proteins encoded by the selected genes, *TLR3, CGAS, TBK1, IKBKE*, *TMEM173* and the previously genotyped *IFNs* genes, interplay with each other.

**Figure _2_SuppInfo.pdf** TLR3*, CGAS, TBK1, IKBKE*, and *TMEM173* pair-wise interactions.

**Figure _3_SuppInfo.pdf** Global view of pair-wise interactions between *TLR3, CGAS, TBK1, IKBKE*, *TMEM173,* and the previously genotyped *IFN* variants*

**Figure _4_SuppInfo.pdf** Pair-wise-interactions exhibited by the two *TMEM* SNPs, when analyzed in interplay with the previously genotyped *IFN* variants.*

**Figure _5_SuppInfo.pdf** Pair-wise-interactions exhibited by the four *CGAS* SNPs, when analyzed in interplay with the previously genotyped *IFN* variants.*

**Figure _6_SuppInfo.pdf** Global view of pair-wise interactions between *IFNs* variants showing the r^2^ value for the SNPs mapping at the same locus on the Chromosome 9 (9p21.3) *

*****The interaction networks were generated by the web-based visualization software VisANT **(**[**http://visant.bu.edu**](http://visant.bu.edu)**)** (1) and personalized with Microsoft PowerPoint.

1. Hu, Z. (2005) VisANT: data-integrating visual framework for biological networks and modules. Nucleic Acids Res vol. 33, pp. W352–W357.
